# GWAS of 89,283 individuals identifies genetic variants associated with self-reporting of being a morning person

**DOI:** 10.1038/ncomms10448

**Published:** 2016-02-02

**Authors:** Youna Hu, Alena Shmygelska, David Tran, Nicholas Eriksson, Joyce Y. Tung, David A. Hinds

**Affiliations:** 1 23andMe, Inc., 899 W Evelyn Avenue, Mountain View, California 94043 USA; 2 Department of Biological Sciences, San Jose State University, San Jose, California 95112 USA

## Abstract

Circadian rhythms are a nearly universal feature of living organisms and affect almost every biological process. Our innate preference for mornings or evenings is determined by the phase of our circadian rhythms. We conduct a genome-wide association analysis of self-reported morningness, followed by analyses of biological pathways and related phenotypes. We identify 15 significantly associated loci, including seven near established circadian genes (rs12736689 near *RGS16*, *P*=7.0 × 10^−18^; rs9479402 near *VIP*, *P*=3.9 × 10^−11^; rs55694368 near *PER2*, *P*=2.6 × 10^−9^; rs35833281 near *HCRTR2*, *P*=3.7 × 10^−9^; rs11545787 near *RASD1*, *P*=1.4 × 10^−8^; rs11121022 near *PER3*, *P*=2.0 × 10^−8^; rs9565309 near *FBXL3*, *P*=3.5 × 10^−8^. Circadian and phototransduction pathways are enriched in our results. Morningness is associated with insomnia and other sleep phenotypes; and is associated with body mass index and depression but we did not find evidence for a causal relationship in our Mendelian randomization analysis. Our findings reinforce current understanding of circadian biology and will guide future studies.

## Abstract (web summary)

Circadian rhythms and related behaviours vary across individuals. Here, a large genome-wide association study reveals common single nucleotide variants influencing whether an individual reports as being a ‘morning person' by identifying 15 significant loci, including 7 near known circadian genes.

## Graphical abstract

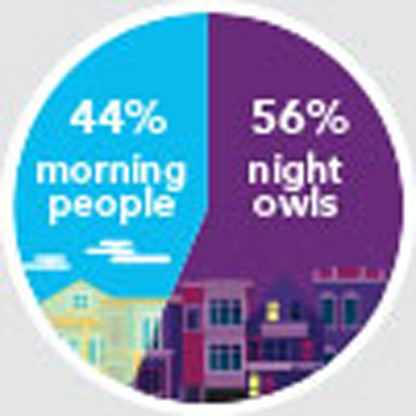

Amorning person prefers to rise and rest early, whereas a night person would choose a cycle later in the day. Chronobiology, the study of such differences (or chronotypes), began with Kleitman^1^ suggesting their existence and Horne and Ostberg^2^ designing a questionnaire for their definition. Morningness is governed by a circadian rhythm mediated by the suprachiasmatic nucleus (SCN) in the hypothalamus. The SCN is a network of cellular oscillators that are synchronized in response to light input received from the human retina^3^. Differences in circadian rhythm have been associated with medically relevant traits such as sleep^4^, obesity^5^ and depression^6^.

Most genetic studies of circadian rhythm have been conducted on model organisms, beginning with the discovery of a first circadian clock gene *per* in Drosophila and *CLOCK* in mice (Supplementary Table 1). Human linkage studies have implicated *PER2* in familial advanced sleep phase syndrome^7^ and candidate gene studies^8^,^9^ have found others. However, study sizes have been small and findings are not robust^10^. Furthermore, few genome-wide association studies (GWAS) have been successful in identifying significant associations^11^,^12^,^13^.

We analysed genetic associations of self-reported morningness using the 23andMe cohort (*n*=89,283) and identified a total of 15 genome-wide significant loci with seven of them close to well-established circadian genes such as *PER2*. We performed pathway analyses and found both circadian and phototransduction pathways enriched in our results. In addition, we observed significant associations between morningness and body mass index (BMI) and depression in our cohort but found no evidence to support a causal relationship in a Mendelian randomization (MR) analysis.

## Results

### Descriptions of GWAS study and cohort

We conducted a GWAS of self-reported morningness in the 23andMe participant cohort^14^, across a total of ∼8 million genotyped or imputed polymorphic sites. Morningness was defined by combining the highly concordant responses (Cohen's Kappa=0.95, *P*<1.0 × 10^−200^) to two web based survey questions that ask if the individual is naturally a morning or night person (Supplementary Table 2). Among 135,447 who answered at least one survey, 75.5% were scored as morning or night persons. Individuals who provided neutral (*n*=32,842) or discordant responses (*n*=309) were removed (Supplementary Table 9). We did not find differences in age, gender or principal components (PCs; all *P*>0.01) when comparing individuals who provided discordant responses versus individuals who gave concordant responses (*n*=12,442). We included individuals of European ancestry who had consented for research, and related individuals were removed from analysis (Methods section). Morningness is significantly associated with gender (*P*=4.4 × 10^−77^), with a prevalence of 39.7% in males and 48.4% in females. Its prevalence increases with age (*P*<1.0 × 10^−200^): 24.2% of those under 30-years-old prefer mornings compared with 63.1% of those over 60. This age trend is consistent with previous reported observations^15^.

Table 1 (together with Supplementary Table 2) shows the marginal association between morningness and other sleep phenotypes, BMI and depression (defined in Supplementary Table 3). Morning persons are significantly less likely to have insomnia (12.9 versus 18.3%, odds ratio (OR)=0.66, *P*=2.4 × 10^−74^). They are also less likely to require >8 h of sleep per day (OR=0.67, *P*=1.1 × 10^−72^), to sleep soundly (OR=0.74, *P*=8.5 × 10^−50^), to sweat while sleeping (OR=0.8, *P*=1.0 × 10^−23^) and to sleep walk (OR=0.77, *P*=4.7 × 10^−10^). Morningness is also associated with lower prevalence of depression (OR=0.64, *P*=1.1 × 10^−128^, Supplementary Table 11). Morning persons are less prevalent in extreme BMI groups, namely the underweight (≤18.5) and the obese (≥30) group (Table 1, Supplementary Fig. 2). However, we found that after for adjusting for age and sex, the prevalence of morning persons decreases monotonically across increasing BMI categories (Supplementary Table 11).

**Figure 1:**
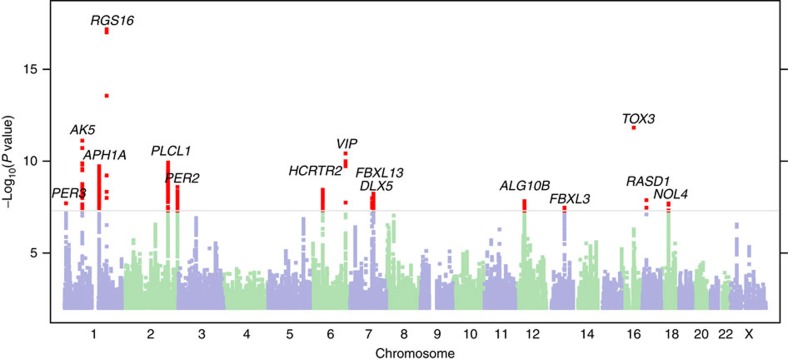
Manhattan plot of the GWAS of being a morning person. The grey line corresponds to *P*=5.0 × 10^−8^, and the results above this threshold are shown in red. Gene labels are annotated as the nearby genes to the significant SNPs.

We included age, sex and the first 5 PCs in a logistic regression model and computed likelihood ratio tests for association of each genotyped or imputed marker with morningness. Association test results were adjusted for a genomic inflation factor of 1.21 (Supplementary Data 1). For an equivalent study of 1,000 cases and 1,000 controls, the genomic inflation factor (known as *λ*_1,000_ (ref. 16)) would be 1.005. The Manhattan plot (Fig. 1) shows 15 morningness-associated regions with genome-wide significance (*P*<5 × 10^-8^). Table 2 categorizes their index single nucleotide polymorphisms (SNPs) by nearby genes. We used Haploreg^17^, a web based computational tool to explore chromatin states, conservations and regulatory motif alterations using public databases, to understand the possible functional roles of these index SNPs (Supplementary Table 16 and Supplementary Data 2).

### Genetic association analyses

Seven loci are near well-established circadian genes. rs12736689 (*P*=7.0 × 10^−18^) is in strong linkage disequilibrium (LD) (*r*^2^=0.89) with the nonsynonymous variant rs1144566 (H137R) of nearby gene *RGS16* (Supplementary Fig. 3), a G protein signalling regulator that inactivates G protein alpha subunits. *RGS16* knock-out mice were shown to have a longer circadian period^18^. rs9479402 (*P*=3.9 × 10^−11^) is 54 kb upstream of *VIP* (Supplementary Fig. 4), a key neuropeptide in the SCN (ref. 19). Its intracerebroventricular administration was found to prolong rapid eye movement sleep in rabbits^20^. rs55694368 (*P* =3.9 × 10^−11^) is 120 kb upstream of *PER2* (Supplementary Fig. 5), which has been associated with human familial advanced sleep phase syndrome^7^. This SNP is located in a DNAse hypersensitive site (DHS) for five cell types, including pancreas adenocarcinoma, B-lymphocyte (GM12891 and GM12892), medulloblastoma and CD4+ cells (Supplementary Table 16B), and alters five regulatory motifs. (See details in Supplementary Tables 16 and 17). rs35833281 (*P*=3.7 × 10^−9^) is 18 kb downstream of *HCRTR2*, or orexin receptor type 2 (Supplementary Fig. 6) and alters eight regulatory motifs (Supplementary Table 16). Mutations in *HCRTR2* have been linked to narcolepsy in dogs and humans^21^,^22^. This SNP rs35833281 is in partial LD with two SNPs (*r*^2^=0.25 for rs2653349 and *r*^2^=0.31 for rs3122169) on *HCRTR2* that were suggested to associate with cluster headache and narcolepsy^23^. These SNPs were also but less significantly associated with morningness (*P*=3.6 × 10^−7^ for rs2653349 and *P*=1.8 × 10^−6^ for rs3122169). rs11545787 (*P*=1.4 × 10^−8^) is a 3′UTR variant of *RASD1* (Supplementary Fig. 7), a G protein signaling activator^24^ and is a promoter histone mark for six cell types (H1, umbilical vein endothelial, B-lymphocyte, lung fibroblasts, skeletal muscle myoblasts and epidermal keratinocyte), in a DHS for seven cell types (skeletal muscle myoblasts, fibroblast, hepatocytes, medulloblastoma, epidermal melanocytes, pancreatic islets and fibroblasts) (Supplementary Table 16). In fact, deletion of *RASD1* has been shown to result in a reduction of photic entrainment in mouse^25^. rs11121022 (*P*=2.0 × 10^−8^), known to alter three regulatory motifs, is 8 kb downstream of *PER3* (Supplementary Fig. 8), which affects the sensitivity of the circadian system to light^26^ and is involved in sleep/wake activity^27^. Variation in *PER3* has also been associated with delayed sleep syndrome and extreme diurnal preference^28^. A recent smaller study^13^ identified another SNP (rs228697) as a significant association with diurnal preference; however, this SNP is much less significant in our GWAS (*P*=5.3 × 10^−5^) and is in low LD with our index SNP rs11121022 (*r*^2^=0.08). rs9565309 (*P*=3.5 × 10^−8^), locating in a DHS for 16 cell types (Supplementary Table 16, Supplementary Data 2), is an intronic variant of *CLN5* and is ∼2 kb downstream of *FBXL3* (Supplementary Fig. 9), part of the F-box protein family, which ubiquitinates light-sensitive cryptochrome proteins *CRY1* and *CRY2*, and mediates their degradation^29^. Mutant *FBXL3* mice were shown to have an extended circadian period^30^.

We found four additional SNPs are linked to genes that are plausibly circadian by literature review for reported potential connections between the genes and circadian rhythms. rs1595824 (*P*=1.2 × 10^−10^) is an intronic variant of *PLCL1* (Supplementary Fig. 10), which is expressed predominantly in the central nervous system and binds to the γ-aminobutyric acid (GABA) type A receptor. rs12965577 (*P*=2.1 × 10^−8^) is an intronic variant of *NOL4* (Supplementary Fig. 13), one of 20 genes with the most significant changes in expression in mice with a knock-in mutation in the α1 subunit of the GABA(A) receptor^31^. As most SCN neuropeptides are colocalized with GABA (ref. 32) and most SCN neurons have GABAergic synapses^33^, it is possible that *PLCL1* and *NOL4* have circadian roles. rs34714364 (*P*=2.0 × 10^−10^), an enhancer histone mark, known to alter 11 regulatory motifs, a synonymous variant of gene *CA14*, is 3 kb away from *APH1A* (Supplementary Fig. 11). *APH1A* encodes a component of the γ-secretase complex which cleaves the β-amyloid precursor protein^34^, and is regulated by orexin and the sleep-wake cycle^35^. This relationship of γ-secretase and sleep-wake cycle suggests a circadian role for *APH1A*, but this region has many genes and further work is needed to verify this hypothesis. rs3972456 (*P*=6.0 × 10^−9^), locating in a DHS for 8 cell types and known to alter three regulatory motifs, is an intronic variant of *FAM185A* and is 16 kb away from *FBXL13* (Supplementary Fig. 12). *FBXL13* also encodes a protein-ubiquitin ligase and may have a circadian role similar to *FBXL3*.

The relationship of the remaining loci to circadian rhythm is less clear. rs12927162 (*P*=1.6 × 10^−12^) is 104 kb upstream of *TOX3* (Supplementary Fig. 14), a gene associated with restless leg syndrome^36^. The regional plot around rs12927162 shows that the next best SNP only has a *P* value of 10^−6^. This SNP alters a POU2F2 motif, but we found no other functional annotation, and additional work is needed to verify this association. Notably, this SNP is not in LD (*r*^2^ =1.2 × 10^−4^) with the reported SNP rs3104767 for restless leg syndrome^36^ and SNPs rs3803662 and rs4784227 for breast cancer^37^,^38^ (Supplementary Table 12). And none of these SNPs have strong association with morningness (*P*>0.01). rs10493596 (*P* =8.0 × 10^−12^) is 21 kb upstream of *AK5* (Supplementary Fig. 15), a gene that regulates adenine nucleotide metabolism expressed only in the brain^39^. rs2948276 (*P*=1.1 × 10^−8^, Supplementary Fig. 16), known to locate in a DHS for three cell types and alter four motifs, is 192 kb downstream of *DLX5* and 118 kb upstream of *SHFM1*, a region linked to split hand/foot malformation. rs6582618 (*P*=1.5 × 10^−8^) is 2 kb upstream of *ALG10B* (Supplementary Fig. 17), a gene with a role in regulation of cardiac rhythms^40^.

For the above significant loci, we performed stepwise conditional analyses to identify potential additional associated variants that are within 200 kb of the index SNPs. We iteratively added new SNPs into the model until no SNP had *P*<1.0 × 10^−5^. We identified one new SNP (Supplementary Table 6) respectively for the locus close to *VIP* (rs62436127, *P*=1.6 × 10^−6^), *APH1A* (rs10888576, *P*=5.0 × 10^−6^) and *PER2* (rs114769095, *P*=9.7 × 10^−6^). Accounting for the ∼15,000 total SNPs that we included in our conditional analysis, the secondary hit around *VIP* is significant (*P*<3.3 × 10^−6^) but the other two are not.

We tested for interaction between these SNPs and age, gender, BMI, alcohol abuse, nicotine abuse and current caffeine use (see Supplementary Table 1 for definitions). First, we added each covariate into the null model of morning person versus age, sex and five PCs. Effects of BMI (OR=0.97 kg^−1^ m^−2^, *P*=1.0 × 10^−125^) and nicotine abuse (OR=0.71, *P*=3.9 × 10^−41^) were significant (Supplementary Table 7A). We then added each SNP into each new null model. Effect sizes were not substantially altered, though *P* values generally became less significant, consistent with the degree of reduction in sample size for these covariates (Supplementary Table 7B). We also added interaction terms (Supplementary Table 7C) for the significant SNPs and covariates to each model and found none that would be significant after accounting for multiple testing. In addition, we estimated SNP effects in three age groups (<45, 45–60 and >60) and found them consistent across these groups (*P*>0.01, Supplementary Table 7D). We also estimated 21% (95% confidence interval (CI; 13%, 29%)) of the variance of the liability of morningness can be explained by genotyped SNPs, using Genome-wide Complex Trait Analysis (GCTA) (ref. 41) on a random subset of 10,000 samples due to computational constraints. Finally, we included the ‘neutral' responders and defined a chronotype phenotype to describe morning, neutral and night person and then performed GWAS on it using a linear model with adjustment of age, sex and top five PCs. We found the results are largely similar to our morning-person GWAS. Detailed comparison (Supplementary Fig. 18) shows that in the chronotype GWAS the loci near *FBXL3*, *RASD1* and *NOL4* were no longer genome-wide significant. Two additional loci reached genome-wide significance at rs2975734 in *MSRA* (Supplementary Fig. 19) and rs9357620 in *PHACTR1* (Supplementary Fig. 20, Supplementary Table 10). *MSRA* has been related to circadian rhythms in Drosophila^42^. *PHACTR1* has not been reported to relate to circadian rhythms but has known associations with myocardial infarction^43^.

### Pathway analyses

We used MAGENTA (ref. 44) to evaluate whether any biological pathways were enriched in our GWAS results (Table 3). The top three pathways are circadian related and share four genes: *PER2* (gene based *P* value=1.6 × 10^−8^), *ARNTL* (*P*=1.2 × 10^−3^), *CRY1* (*P*=3.7 × 10^−3^) and *CRY2* (*P*=5.2 × 10^−3^). In addition, *PER3* (*P*=1.4 × 10^−7^), in the KEGG circadian rhythm pathway, and *FBXL3* (*P*=9.4 × 10^−8^), in the REACTOME circadian clock pathway, have strong effects and were implicated in our GWAS. Other circadian genes also contribute to the enrichment of circadian pathways, but less significantly (Table 3). The BH4 related pathway (gene set *P*=3.1 × 10^−3^) has a major role in the biosynthesis of melatonin, serotonin and dopamine, which are important hormones involved in circadian rhythm regulation and brain function. The phospholipase C (PLC) β-mediated events pathway (*P*=4.3 × 10^−3^) includes *GNAO1* (*P*=6.2 × 10^−4^), *GNAI3* (*P*=5.5 × 10^−3^), *GNAT1* (*P*=1.0 × 10^−2^) and many other G protein related genes involved in visual phototransduction. *GNAT1* is related to night blindness^45^ and *GNAI3* is known to interact with *RGS16* (ref. 46). Interestingly, *RGS16* is close to our GWAS top hit. This pathway also includes *PRKAR2A* (*P*=1.4 × 10^−3^) and *PRKACG* (*P*=0.047), which relate to cAMP dependent protein kinase A, known to regulate critical processes in the circadian negative feedback loops^47^. Notably, except for the KEGG circadian rhythm pathway, which has a false discovery rate 0.06, all other associated pathways have false discovery rate >0.2, meaning the statistical evidence of the association is not strong.

We assessed correlations between morningness and related phenotypes with adjustment for potential confounders by regression with covariates for age, gender and ancestry (Table 4). The covariate-adjusted odds of having insomnia for morning people is 55% of that for night people (*P*=1.5 × 10^−140^) and the adjusted odds of having sleep apnoea for morning people is 64% of that for night people (*P*=4.0 × 10^−54^). Morning people are also less likely to require >8 h of sleep (OR=0.69, *P*=6.3 × 10^−53^), to sleep soundly (OR=0.81, *P*=6.8 × 10^−24^), to have restless leg syndrome (OR=0.71, *P*=4.1 × 10^−15^) and sweat while sleeping (OR=0.90, *P*=7.9 × 10^−6^) after adjusting for covariates. Sleepwalking and actual amount of sleep do not correlate with morningness (*P*>0.1) in the full model. These associations are consistent with previous studies of insomnia^48^, sleep apnoea^49^ and sleep needed^50^. We calculated the association between the 15 GWAS identified SNPs and these eight sleep phenotypes (Supplementary Table 4) but found no significant associations. In addition, we looked up the SNPs and their proxies in the latest BMI GWAS from the GIANT consortium^51^ and the latest major depressive disorder GWAS from the CONVERGE Consortium^52^. But we did not find significant associations (Supplementary Table 4B,C).

We examined previously identified associations of morningness with BMI (ref. 5) and depression^6^. We found that that the covariate-adjusted odds for morning people to report depression is 61% of that for night people (*P*=3.5 × 10^−138^), and the average BMI for morning people is 0.99 kg m^−2^ lower (*P*=1.6 × 10^−125^), adjusting for covariates (Table 4). We also calculated the association between the 15 significant GWAS SNPs and depression and BMI but found no significant associations (Supplementary Table 4).

### MR analyses

We used a MR approach to find evidence in support of a causal relationship of morningness with BMI. We first calculated a morningness genetic risk score by summing the risk alleles of the seven circadian related SNPs weighted by their effects, then regressed morningness or BMI against this instrument variable while adjusting for covariates (age, sex and top five PCs), and consequently estimated the ratio of the covariate-adjusted genetic effect for morningness to that for BMI (Methods section). Morningness is highly correlated (F statistic=19.0, *P*=2.1 × 10^−80^ in the linear regression model and *P*=1.5 × 10^−79^ in the logistic regression) with the genetic risk, but BMI (*P*=0.43) is not (Table 5). We further estimated the transferred genetic effect, that is, the effect from genetically elevated chance of being a morning person on BMI as −0.34 kg m^−2^(95% CI: (−0.99, 0.96), *P*=0.91) per unit increase of probability of being a morning person. Similarly, we found that depression is not significantly correlated with morningness genetic risk (*P*=0.10). We estimated a non-significant transferred genetic effect of morningness on depression: the probability of depression decreases by 0.07 (95% CI: (−0.10, 0.11), *P*=0.18) per unit increase of probability of being a morning person. Thus, we did not find evidence for morningness to be protective of depression or high BMI. Notably, the power of the MR analysis is governed by the strength of the correlation between morningness and its genetic risk as well as the magnitude of the transferred genetic effect of morningness on BMI or depression. We ran simulations (Methods section) to assess the power for our MR and found that our current sample sizes, though large by conventional standards, only lead to moderate power in our MR analysis of morningness and BMI and depression (Supplementary Table 8). If the observed correlation is entirely causal, our analysis has only ∼40% power. Our reported lack of statistical evidence in our MR analysis could be due to constrained study power.

We also conducted an MR analysis of BMI on morningness. We retrieved the morningness GWAS results for a set of 28 previously reported BMI associated SNPs (ref. 53) and found rs1558902, an intronic variant of *FTO,* had some evidence for association with morningness (*P*=6.0 × 10^−6^, Supplementary Table 5). We then calculated a BMI genetic risk with this set of SNPs using the previously reported effect sizes. It is highly correlated with BMI (F statistic=47.4, *P*<1.0 × 10^−200^) but we found it to be uncorrelated with morningness (*P*=0.26) and found no support for a causal relationship (transferred genetic effect=0.0029, 95% CI: (−0.0059, 0.006), *P*=0.35). Our power calculation (Supplementary Table 8) shows that this MR analysis is well powered (∼80%) to show evidence of a causal relationship between BMI and morningness, assuming the observed correlation is entirely causal.

## Discussion

We identified many loci significantly associated with morningness but were unable to find clear genetic associations in our GWAS analysis of related sleep phenotypes, such as insomnia, sleep apnoea, sleep needed, sleeping soundly and sweating while sleeping. These sleep phenotypes may be more genetically heterogeneous and our current sample sizes, while large by most standards, maybe still be too small for discoveries. It is also possible that environmental factors mediate the association between morningness and these sleep phenotypes. These other phenotypes may also be more subject to possible self-reporting bias. We assessed morningness with simple questions and did not consider light exposure, season, geography and other factors, it is possible that better results would be obtained from using more-detailed surveys (such as the standard Horne–Ostberg questionnaire^2^). We have also considered the effect of smoking, drinking and caffeine consumption in our analysis but with limited thoroughness for these phenotypes. More-detailed phenotyping would be desirable for future studies, though GWAS typically do not adjust for such factors. An analysis including more refined estimates of these covariates would yield more accurate estimates of effect sizes and could reveal information about mechanism, if some associations with sleep phenotypes are mediated by these other behaviours.

For known circadian genes such as *DEC1*, *DEC2*, *BMAL1*, *CRY1* and *CRY2*, we did not find signals that were genome-wide significant. Specifically, within 100 kb windows of each gene, we had 1,712 SNPs for *DEC1* with a minimum *P* value 0.01; we had 689 SNPs for *DEC2* with a minimum *P* value 7.8 × 10^−4^; we had 835 SNPs for *BMAL1* and a minimum *P* value of 1.0 × 10^−6^; we had 804 SNPs for *CRY1* and a minimum *P* value of 4.4 × 10^−6^; we have 504 SNPs for *CRY2* and a minimum *P* value of 8.9 × 10^−6^. Some of these genes may have a less important role in morningness, or may not have genetic variation that could be identified by GWAS. However, the associations in *BMAL1*, *CRY1* and *CRY2* are suggestive and additional data may confirm signals in these genes.

Another large-scale genetic study of chronotype^54^ using the UK Biobank has recently been completed, with results largely consistent with our own. Specifically, that study reports genome-wide-significant loci at *RGS16*, *AK5*, *PER2* and *HCRTR2*, as well as near *FBXL13* and *APH1A*. Further work will be needed to assess replication of other loci not genome-wide significant in both studies.

Our MR analysis did not provide evidence for a causal relationship of morningness on BMI or depression. We have checked MR assumptions (Supplementary Tables 13–15). The F statistics is 19.0 in the linear regression of morning person. Since morningness is binary, we calculated a generalized coefficient of determination for logistic regression: Nagelkerke's *R*^2^=0.0056. Without direct translation between the *R*^2^ and the F statistics, we assumed that this small scale of *R*^2^ could indicate our instrument is weak and our MR analysis could be underpowered. We verified that PCs are associated with both risk and outcomes (Supplementary Table 13A), so we have adjusted for them in our MR analysis (Supplementary Table 13A). In addition, we found the preference of sweet foods (effect=0.147, *P*=7.1 × 10^−3^, Supplementary Table 14) is moderately associated with morning-person genetic risk (OR=0.15, *P*=7.1 × 10^−3^) and BMI (OR=1.009 kg^−1^ m^−2^, *P*=1.5 × 10^−10^, Supplementary Table 14A) and depression (OR=1.24, *P*=6.9 × 10^−27^). We hence included the preference of sweet foods in our MR analysis but found no changes in our conclusion (*P*>0.01).

In addition, we also checked for assumptions in our MR analysis of BMI on morningness (Supplementary Tables 13–15). We found PCs are associated with BMI risk and morning person (Supplementary Table 13B). We also identified current caffeine use (Supplementary Table 14B) is associated with BMI genetic risk (effect=10.5 kg^−1^ m^−2^, *P*=1.3 × 10^−6^) and morning person (effect=18.2, *P*=4.5 × 10^−11^). Adjusting for PCs and current caffeine use did not lead to change to our result (Supplementary Table 15B). Our MR analysis could not rule out canalization or developmental compensation, by which individuals adapt in response to genetic change in a way that the expected effect of the change is reduced^55^. Our analysis also did not test for non-linear relationships between the phenotypes.

Among BMI risk SNPs, we found an *FTO* variant strongly correlated with morningness (Supplementary Table 5). Our MR analysis using a BMI genetic risk score as an instrument variable did not find evidence to support a more general effect of BMI genetic risk on morningness. There may be pleiotropy specifically at the *FTO* locus, instead of a more general casual effect of BMI. Moreover, their strong association may reflect effects of other factors, such as environment, socioeconomics, personality or other genetic variables through independent mechanisms.

## Methods

### 23andMe cohort

Participants in the 23andMe cohort were customers of 23andMe, Inc., a personal genetics company, who had been genotyped as part of the 23andMe Personal Genome Services. DNA extraction and genotyping were performed on saliva samples by the National Genetics Institute (NGI), a Clinical Laboratory Improvement Amendments (CLIA)-certified clinical laboratory and subsidiary of the Laboratory Corporation of America. Samples were genotyped on one of two platforms. About 35% of the participants were genotyped on the Illumina HumanHap550+ BeadChip platform, which included SNPs from the standard HumanHap550 panel augmented with a custom set of ∼25,000 SNPs selected by 23andMe. Two slightly different versions of this platform were used, as previously described^14^. The remaining 65% of participants were genotyped on the Illumina HumanOmniExpress+ BeadChip. This platform has a base set of 730,000 SNPs augmented with ∼250,000 SNPs to obtain a superset of the HumanHap550+ content, as well as a custom set of ∼30,000 SNPs. Every sample that did not reach a 98.5% call rate for SNPs on the standard platforms was reanalyzed. Individuals whose analyses repeatedly failed were contacted by 23andMe customer service to provide additional samples.

We collected phenotypes by inviting participants to login in www.23andme.com to answer surveys that are either comprehensive ones with multiple questions on a subject matter or quick questions. We defined phenotypes by combining the answers to questions on the same subject. For example, as shown in Supplementary Table 2, our morning person phenotype definition is from combining the answers to two questions that asking if the participant is naturally a night person or morning person. For each question, we classify the answer as night person, morning person or missing. Then if one answer is missing, we use the other answer as the phenotype value, and if one answer is morning person but the other is night person, we treated the phenotype value as missing. Similarly, we used appropriate combination rules to derive other phenotypes from multiple survey questions (see more in Supplementary Table 2).

The study protocol and consent form were approved by the external Association for the Accreditation of Human Research Protection Programs-accredited Institutional Review Board, Ethical & Independent Review Services. For a small number of participants (*n*=167) under the age of 18 years, consent was provided by a parent, guardian or legally authorized adult.

### GWAS analysis for 23andMe European samples

For our standard GWAS, we restricted participants to a set of individuals who have >97% European ancestry, as determined through an analysis of local ancestry via comparison to the three HapMap 2 populations^56^. A maximal set of unrelated individuals was chosen for the analysis using a segmental identity-by-descent estimation algorithm^57^. Individuals were defined as related if they shared >700 cM identity-by-descent, including regions where the two individuals share either one or both genomic segments identical-by-descent. This level of relatedness (roughly 20% of the genome) corresponds approximately to the minimal expected sharing between first cousins in an outbred population.

Participant genotype data were imputed against the August 2010 release of 1,000 Genomes reference haplotypes^58^. First, we used Beagle^59^ (version 3.3.1) to phase batches of 8,000–9,000 individuals across chromosomal segments of no >10,000 genotyped SNPs, with overlaps of 200 SNPs. We excluded SNPs with minor allele frequency<0.001, Hardy–Weinberg equilibrium *P*<10^−20^, call rate<95%, or with large allele frequency discrepancies compared with the 1,000 Genomes reference data. We identified the discrepancies by computing a 2 × 2 table of allele counts for the European 1,000 Genomes samples and 2,000 randomly sampled 23andMe customers with European ancestry and excluded SNPs with *χ*^2^ test *P* value <10^−15^. We then assembled full-phased chromosomes by matching the phase of haplotypes across the overlapping segments. We imputed each batch against the European subset of 1,000 Genomes haplotypes using Minimac (2011-10-27)^60^, using five rounds and 200 states for parameter estimation.

For the non-pseudoautosomal region of the X chromosome, males and females were phased together in segments, treating the males as already phased; the pseudoautosomal regions were phased separately. We assembled fully phased X chromosomes, representing males as homozygous pseudo-diploids for the non-pseudoautosomal region. We then imputed males and females together using Minimac as with the autosomes.

For morning and night person comparisons, we computed association test results by logistic regression assuming additive allelic effects. For tests using imputed data, we used the imputed dosages rather than best-guess genotypes. We used covariates age, gender, and the top five PC to account for residual population structure. The GWAS association test *P* values were computed using a likelihood ratio test. Results for the X chromosome are computed similarly, with men coded as if they were homozygous diploid for the observed allele.

Imputed results were computed for 7,381,496 SNPs having an average imputation *r*^2^>0.5 and a minimum within-batch *r*^2^>0.3, and removing SNPs with evidence of a strong batch effect (*P*<10^−50^), measured by ANOVA of dosages versus batches. For genotyped SNPs, we identified 854,959 SNPs with a minor allele frequency >0.1%, call rate >90%, Hardy–Weinberg *P*>10^−20^ in European 23andMe participants and *P*>10^−50^ for an effect of genotyping date on allele frequency. To create a single merged result set, for 806,041 SNPs with both imputed and genotyped results passing these quality filters, we selected the imputed result. After applying these filters and removing a small number of results that did not converge, we were left with association test results for 7,427,422 SNPs.

### Pathway analysis of morningness

We first downloaded a database of canonical pathways of 1,320 biologically defined gene sets^61^, then used gene set enrichment analysis^61^, implemented in MAGENTA (ref. 44), on our morningness GWAS results. MAGENTA first assigns SNPs to a gene within 110 kb upstream and 40 kb downstream of transcript boundaries. The most significant SNP in this gene is then adjusted for confounders (gene size, SNP density and LD) in a regression framework to obtain a score for each gene. Then genes are then ranked according to their scores and then a gene set enrichment analysis-based approach was used to test whether predefined sets of functionally related genes are enriched for genes associated with morning person, more than would be expected by chance. MAGENTA counts the number of genes (enrichment score) with scores ranking above the 95th percentile. To evaluate the significance of each pathway, MAGENTA randomly sample 10,000 gene sets from the genome that are of identical size to the pathway and compare the observed enrichment score to the resampled enrichment score. To adjust for multiple testing, it estimates the false discovery rate by comparing the observed normalized enrichment score to all resampled normalized enrichment score.

### Relationship of morningness and other phenotypes

For binary phenotypes such as insomnia, sleep apnoea, we used a logistic regression to estimate the effect of morningness after adjusting for age, sex and top five PCs. For the continuous phenotype BMI, we used a linear regression model instead. Morningness is part of sleep and its aetiology intertwines with other sleep phenotypes, so it is difficult to dissect the causal relationship. But BMI and depression are not directly involved in sleep, we can treat the genetic risk of being a morning person as an instrument variable for causal inference. We calculated a morningness genetic risk by averaging the genotypes of the seven SNPs that are close to known circadian genes weighted by their log odds ratio. Then we carried out a MR analysis to evaluate the causal role of morningness. The transferred genetic effect on morningness is estimated by dividing the genetic risk effect of the phenotype to that of the morning-person phenotype. For a continuous phenotype (for example, BMI), this genetic risk effect is the change of mean per unit increase of risk estimated by a linear regression. For a binary phenotype (for example, depression and morningness), this genetic risk effect is the change of probability per unit increase of risk estimated by a logistic regression model^62^,^63^. The 95% CI of the transferred genetic effect is estimated using the Bootstrap, where we resampled the cohort for 1,000 times to obtain resampled transferred genetic effects. We then calculated two-sided *P* value by comparing the observed transferred genetic effect and the resampled transferred genetic effects.

For the analysis of the transferred genetic effect of BMI on morningness, we calculated a BMI risk by averaging the genotypes of a total of 28 previously reported BMI loci weighted by their reported effect sizes^53^. We then estimated the transferred genetic effect as the ratio of the genetic risk effect on morningness to that on BMI. The confidence interval and statistical inference is done using Bootstrapping.

In addition, we performed simulations to evaluate the power of our MR analysis^64^. For the analysis of morningness to depression, we kept the genetic risk, age, sex, top five PCs and morning person as observed, and then simulated depression from a Bernoulli distribution with expectation calculated from a logistic regression model. In that model, we included morningness, age, sex and top five PCs as predictors. Their effects were estimated by regressing our observed depression phenotypes against these predictors, except that for morningness, we specified its transferred genetic effect using hypothetical values. Similarly, we simulated BMI using a linear regression with predictors as morningness and other covariates, with the effect of covariates estimated from our data and the transferred genetic effect of morningness hypothetically chosen. The simulation to evaluate the causal role of BMI on morningness was conducted in a similar fashion.

## Additional information

**How to cite this article:** Hu, Y. *et al.* GWAS of 89,283 individuals identifies genetic variants associated with self-reporting of being a morning person. *Nat. Commun.* 7:10448 doi: 10.1038/ncomms10448 (2016).

## Supplementary Material

Supplementary Information Supplementary Figures 1-20 and Supplementary Tables 1-16

Supplementary Data 1 GWAS summary statistics for the top 10,000 SNPs

Supplementary Data 2 Detailed HaploReg results for the functional annotations of significant SNPs

## Figures and Tables

**Table 1:** Demographic characteristics of the GWAS cohort.

	**Morning persons** ***N*** **(% of total)**	**Evening persons** ***N*** **(% of total)**	**Proportion that are morning persons (%)**
Total	38,937 (100.0)	50,346 (100.0)	43.6
			
*Sex*			
Male	19,569 (50.3)	29,713 (59.0)	39.7
Female	19,368 (49.7)	20,633 (41.0)	48.4
			
*Age*			
<30	3,684 (9.5)	11,521 (22.9)	24.2
30–45	8,809 (22.6)	19,470 (38.7)	31.2
45–60	12,295 (31.6)	11,111 (22.1)	52.5
>60	14,149 (36.3)	8,244 (16.4)	63.1
			
*Insomnia*			
No	13,809 (79.1)	14,180 (60.3)	49.3
Yes	3,639 (20.9)	9,348 (39.7)	28.0
			
*Sleep apnoea*			
No	22,827 (89.5)	30,822 (88.9)	42.5
Yes	2,673 (10.5)	3,862 (11.1)	40.9
			
*Sleep needed*			
<8 h	7,549 (56.6)	8,715 (46.4)	46.4
≥8 h	5,782 (43.4)	10,068 (53.6)	36.4
			
*Sound sleeper*			
No	8,772 (49.3)	10,062 (42.0)	46.6
Yes	9,020 (50.7)	13,901 (58.0)	39.4
			
*Restless leg syndrome*			
No	11,877 (92.0)	16,476 (91.3)	41.9
Yes	1,035 (8.0)	1,566 (8.7)	39.8
			
*Sweat while sleeping*			
No	12,809 (72.9)	16,273 (68.3)	44.0
Yes	4,765 (27.1)	7,546 (31.7)	38.7
			
*Sleep walk*			
No	12,145 (92.9)	16,773 (90.9)	42.0
Yes	934 (7.1)	1,681 (9.1)	35.7
			
*Average daily sleep duration*			
<8 h	8,146 (67.6)	11,102 (68.5)	42.3
≥8 h	3,902 (32.4)	5,095 (31.5)	43.4
			
*Depression*			
No	20,217 (77.6)	24,162 (68.8)	45.6
Yes	5,835 (22.4)	10,977 (31.2)	34.7
			
*BMI (kg m^−2^)*			
≤18.5 (Underweight)	609 (1.8)	947 (2.1)	39.1
18.5–25 (Normal)	14,561 (42.9)	19,261 (41.8)	43.1
25–30 (Overweight)	12,803 (35.6)	15,440 (33.5)	45.3
>30 (Obese)	6,677 (19.7)	10,464 (22.7)	39.0

BMI, body mass index.

**Table 2:** Index significant SNPs that are associated with being a morning person.

**Gene context**	**Marker name**	**Chromosome**	**Position**	**SNP quality**	**Alleles (A/B)**	**BAF**	**OR for B allele**	**95% CI**	***P*** **value**
*Genes with well-known circadian role*									
*RGS16* (*RNASEL*)	rs12736689	1	182549729	0.97	C/T	0.97	0.74	(0.69, 0.79)	7.0 × 10^−18^
*VIP*	rs9479402	6	153135339	0.85	C/T	0.99	0.69	(0.62, 0.77)	3.9 × 10^−11^
*PER2*	rs55694368	2	239317692	0.66	G/T	0.07	0.86	(0.81, 0.90)	2.6 × 10^−9^
*HCRTR2* (aka *OX2R*)	rs35833281	6	55021561	0.99	C/G	0.79	0.92	(0.90, 0.95)	3.7 × 10^−9^
*RASD1*	rs11545787	17	17398278	0.88	A/G	0.76	1.08	(1.05, 1.11)	1.4 × 10^−8^
*PER3* (*VAMP3*)	rs11121022	1	7836659	0.98	A/C	0.42	1.07	(1.04, 1.09)	2.0 × 10^−8^
*FBXL3* (*CLN5*)	rs9565309	13	77577027	>0.99	C/T	0.97	1.19	(1.12, 1.26)	3.5 × 10^−8^

*Genes with plausible circadian role*									
*PLCL1*	rs1595824	2	198874006	>0.99	C/T	0.49	1.08	(1.05, 1.10)	1.2 × 10^−10^
*APH1A* (*CA14*)	rs34714364	1	150234657	0.64	G/T	0.17	1.12	(1.08, 1.16)	2.0 × 1 0^−10^
*FBXL13* (*FAM185A*)	rs3972456	7	102436907	0.66	A/G	0.71	0.92	(0.89, 0.94)	6.0 × 10^−9^
*NOL4*	rs12965577	18	31675680	>0.99	A/G	0.34	0.94	(0.92, 0.96)	2.1 × 10^−8^

*Genes with less clear circadian role*									
*TOX3*	rs12927162	16	52684916	0.96	A/G	0.26	0.91	(0.89, 0.94)	1.6 × 10^−12^
*AK5*	rs10493596	1	77726241	>0.99	C/T	0.24	1.09	(1.07, 1.12)	8.0 × 10^−12^
*DLX5* (*SHFM1*)	rs2948276	7	96457119	0.99	A/G	0.18	0.92	(0.89, 0.95)	1.1 × 10^−8^
*ALG10B*	rs6582618	12	38726137	0.92	A/G	0.52	1.07	(1.04, 1.09)	1.5 × 10^−8^

BAF, B allele frequency; CI, confidence interval; SNP, single nucleotide polymorphism; gene context is the gene close to the index SNP; alleles A and B are assigned based on their alphabetical order; OR, odds ratio for the B allele; *P* values have been adjusted for a genomic control inflation factor of 1.21; position is the build hg19 map position of the SNP; SNP quality is *r*^2^ from imputation.

**Table 3:** Top five morningness-associated pathways analysed by MAGENTA.

**Database**	Gene set*	**# Of genes**	**Gene set** ***P*** **value**	**FDR**	**Gene name**	**Gene level** ***P*** **value**†	**Variant**	**Variant specific** ***P*** **value**
KEGG	Circadian rhythm	13	2.0 × 10^−4^	0.059	*PER2*	1.6 × 10^−8^	rs150174970	7.8 × 10^−9^
					*PER3*	1.4 × 10^−7^	rs11121022	2.0 × 10^−8^
					*ARNTL*	1.2 × 10^−3^	rs12805304	9.3 × 10^−6^
					*CRY1*	3.7 × 10^−3^	rs12298001	3.1 × 10^−5^
					*CRY2*	5.2 × 10^−3^	rs7127456	5.5 × 10^−5^
REACTOME	Circadian clock	53	4.0 × 10^−4^	0.20	*PER2*	1.6 × 10^−8^	rs150174970	7.8 × 10^−9^
					*FBXL3*	9.4 × 10^−8^	rs9565309	3.5 × 10^−8^
					*ARNTL*	1.2 × 10^−3^	rs12805304	9.3 × 10^−6^
					*CRY1*	3.7 × 10^−3^	rs12298001	3.1 × 10^−5^
					*CRY2*	5.2 × 10^−3^	rs7127456	5.5 × 10^−5^
					*NCOA6*	1.5 × 10^−2^	rs140865084	1.7 × 10^−4^
					*MEF2C*	2.6 × 10^−2^	rs13155750	1.9 × 10^−4^
					*NR3C1*	3.0 × 10^−2^	rs10482700	1.8 × 10^−4^
					*ATF2*	3.9 × 10^−2^	rs7554 8314	3.1 × 10^−4^
					*SERPINE1*	4.3 × 10^−2^	rs75413959	3.5 × 10^−4^
REACTOME	BMAL1, CLOCK, NPAS2 activates circadian expression	36	1.9 × 10^−3^	0.42	*PER2*	1.6 × 10^−8^	rs150174970	7.8 × 10^−9^
					*ARNTL*	1.2 × 10^−3^	rs12805304	9.3 × 10^−6^
					*CRY1*	3.7 × 10^−3^	rs12298001	3.1 × 10^−5^
					*CRY2*	5.2 × 10^−3^	rs7127456	5.5 × 10^−5^
					*NCOA6*	1.5 × 10^−2^	rs140865084	1.7 × 10^−4^
					*NR3C1*	3.0 × 10^−2^	rs10482700	1.8 × 10^−4^
					*SERPINE1*	4.3 × 10^−2^	rs75413959	3.5 × 10^−4^
REACTOME	Tetrahydrobiopterin BH4 synthesis recycling salvage and regulation	13	3.1 × 10^−3^	0.22	*GCHFR*	2.3 × 10^−2^	rs8036891	2.9 × 10^−4^
					*GCH1*	2.4 × 10^−2^	rs998259	2.0 × 10^−4^
					*AKT1*	3.8 × 10^−2^	rs117725127	3.1 × 10^−4^
					*NOS3*	3.9 × 10^−2^	rs3800779	3.1 × 10^−4^
REACTOME	Phospholipase C−β-mediated events	43	4.3 × 10^−3^	0.52	*GNAO1*	6.2 × 10^−4^	rs2398144	5.5 × 10^−6^
					*PRKAR2A*	1.4 × 10^−3^	rs56411893	3.2 × 10^−5^
					*GNAT2*	5.1 × 10^−3^	rs72705206	6.9 × 10^−5^
					*GNAI3*	5.5 × 10^−3^	rs72705206	6.9 × 10^−5^
					*ADCY3*	8.7 × 10^−3^	rs4665746	5.6 × 10^−5^
					*GNAT1*	1.0 × 10^−2^	rs62263597	1.7 × 10^−4^
					*GNAI2*	1.1 × 10^−2^	rs2282749	1.7 × 10^−4^
					*PRKACG*	4.7 × 10^−2^	rs10118146	3.3 × 10^−4^

FDR, false discovery rate.

^*^The gene set is from the database of canonical pathways of 1,320 biologically defined gene sets (http://www.broadinstitute.org/gsea/msigdb/index.jsp).

^†^For each pathway, we only include genes with a *P* value<0.05.

**Table 4:** Association of morningness and other phenotypes adjusting for age, sex, and 5 PC.

**Other phenotype**	**Sample size**	Effect size*	**95% CI**	***P*** **value**
*Model: logistic regression of the binary phenotype versus morning person, age, sex and top five PCs*				
Insomnia	40,976	OR=0.41	(0.39, 0.42)	<1.0 × 10^−200^
Sleep apnea	60,184	OR=0.64	(0.61, 0.68)	4.0 × 10^−54^
Sleep needed (≥8 h)	32,114	OR=0.69	(0.66, 0.72)	6.3 × 10^−53^
Sound sleeper	41,755	OR=0.81	(0.78, 0.84)	6.8 × 10^−24^
Restless leg syndrome	30,954	OR=0.71	(0.65, 0.77)	4.1 × 10^−15^
Sweat while sleeping	41,393	OR=0.90	(0.86, 0.94)	7.9 × 10^−6^
Sleep walk	31,533	OR=1.05	(0.97, 1.15)	0.24
Average daily sleep duration (≥8 h)	28,245	OR=0.96	(0.91, 1.01)	0.11
Depression	61,191	OR=0.61	(0.59, 0.63)	3.5 × 10^−138^
				
*Model: linear regression of the continuous phenotype versus morning person, age, sex and top five PCs*				
BMI (kg m^−2^)	80,042	Slope=−0.99 (kg m^−2^)	(−1.07, −0.91)	1.6 × 10^−125^

BMI, body mass index; CI, confidence interval; OR, odds ratio; PC, principal component.

^*^In logistic regressions, the effect size is OR that describes the ratio of the odds of answering ‘yes' to binary phenotypes in morning persons to the odds of answering ‘yes' to binary phenotypes in night persons. In linear regression, the slope describes the difference of the average value (for example, BMI) in the morning persons and that in the night persons.

**Table 5:** The relationship between morning person status and BMI and depression, adjusting for covariates.

**Other phenotype**	**Sample size**	Effect size*	**95% CI**	***P*** **value**
*Association with morning-person genetic risk*†				
Morning person	91,967	OR=2.64	(2.39, 2.92)	1.5 × 10^−79^
Depression	61,191	OR=0.92	(0.83, 1.02)	0.10
BMI (kg m^−2^)	80,042	Slope=−0.07	(−0.26, 0.11)	0.43
				
*MR to evaluate transferrable genetic risk of morningness*				
Depression	61,191	Transferred genetic effect =−0.07	(−0.10 0.11)	0.18
BMI	80,042	Transferred genetic effect =−0.34	(−0.99, 0.96)	0.91
				
*Association with BMI genetic risk*‡				
BMI	80,042	Slope=−1.16	(−1.21, −1.11)	<1.0 × 10^−200^
Morning person	91,697	OR=0.99	(0.96, 1.01)	0.26
				
*MR to evaluate the causal relation of BMI to morningness*				
Morning person	91,697	Transferred genetic effect=0.0029	(−0.0059, 0.006)	0.35

BMI, body mass index; CI, confidence interval; MR, mendelian randomization; OR, odds ratio.

^*^Effect size is OR for binary phenotypes and slope (unit increase) for continuous phenotypes in regression analysis. In MR analysis, it is the transferrable genetic effect, which is the ratio of two genetic effects estimated by regressions. The genetic effect is the average difference of prevalence for binary phenotypes and is the average slope for continuous phenotypes.

^†^The morningness genetic risk is calculated by the sum of the risk alleles of the seven genome-wide significant loci that are close to well-known circadian genes, weighted by their effect size estimated in our morning person GWAS (Table 1).

^‡^The BMI genetic risk is calculated by the sum of a set of 28 reported BMI associated alleles (Supplementary Table 3) weighted by the unit change of BMI per additional copy of the associated allele^53^.
