# Supplementary material for: GWAS of 89,283 individuals identifies genetic variants associated with self-reporting of being a morning person
Source: Nat Commun. 2016 Feb 2;7:10448. doi: 10.1038/ncomms10448 (PMC4740817; doi:10.1038/ncomms10448)
Supplement: Supplementary Information — Supplementary Figures 1-20 and Supplementary Tables 1-16 [file ncomms10448-s1.pdf]

Supplementary Figures

Figure S1. QQ plot for the GWAS analysis results of morningness

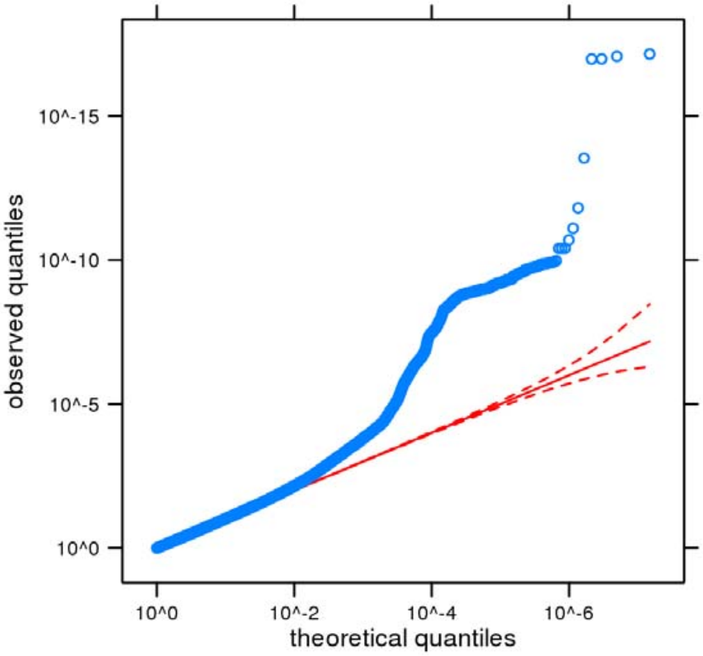

Figure S2. The relation of the fraction of morning persons versus Body Mass Index (15-50 kg m^{-2})

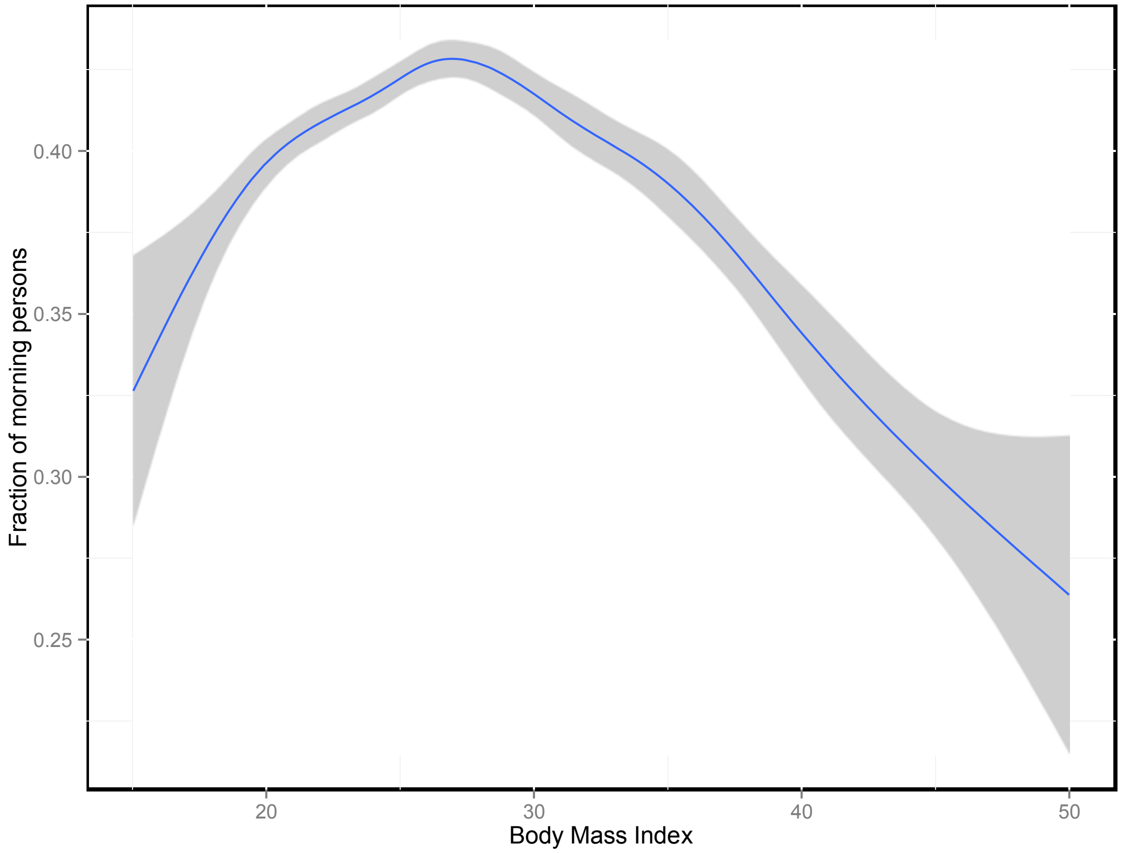

To illustrate the relationship of a binary phenotype (morningness) and a continuous phenotype (BMI), we used the *stat_smooth*() function in ggplot2 [49] with the method parameter as *gam* (generalized additive models with integrated smoothness estimation). The gray band around the smoothed fit is the 95% confidence interval.

Figure S3-Figure S17 Regional association plots of morning person GWAS test statistics versus genomic position in the vicinity of the strongest associations (Table 2). Symbol colors denote linkage disequilibrium with the lead SNP. Open circles 'o' indicate genotyped variants, and the '+' symbols indicate fully imputed variants. Results are in NCBI Build 37 coordinates. The plots are generated with *LocusZoom* [40], using linkage disequilibrium data from the March 2012 release of 1,000 Genome data.

Figure S3. Regional plot for SNP rs12736689, close to gene *RGS16*

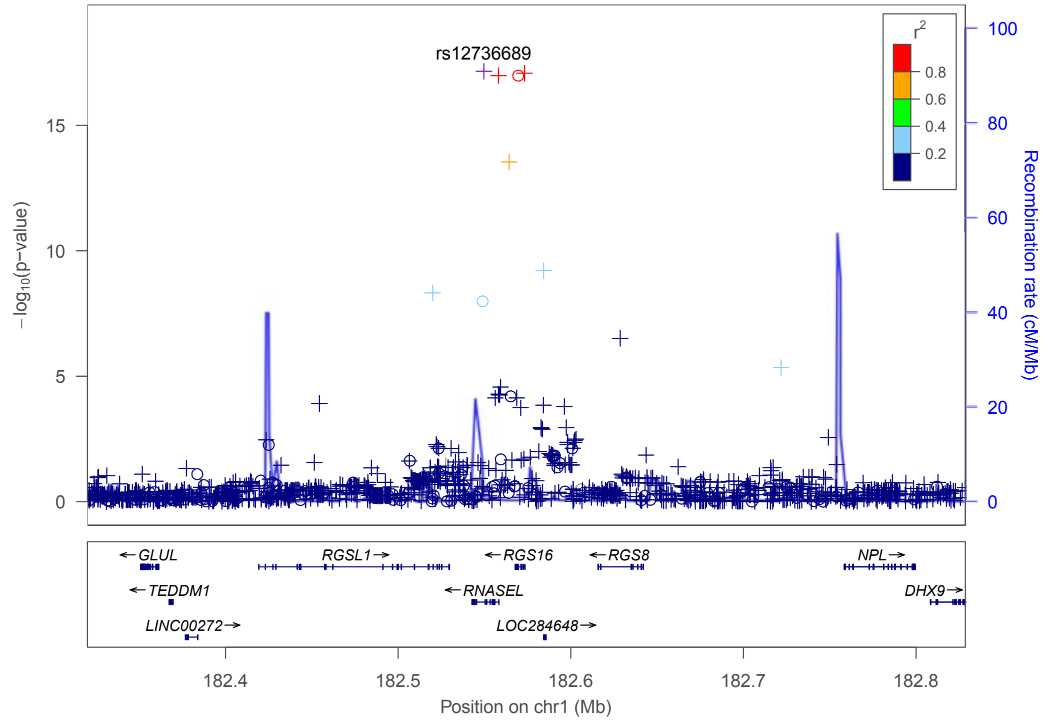

Figure S4. Regional plot for SNP rs9479402, close to gene *VIP*

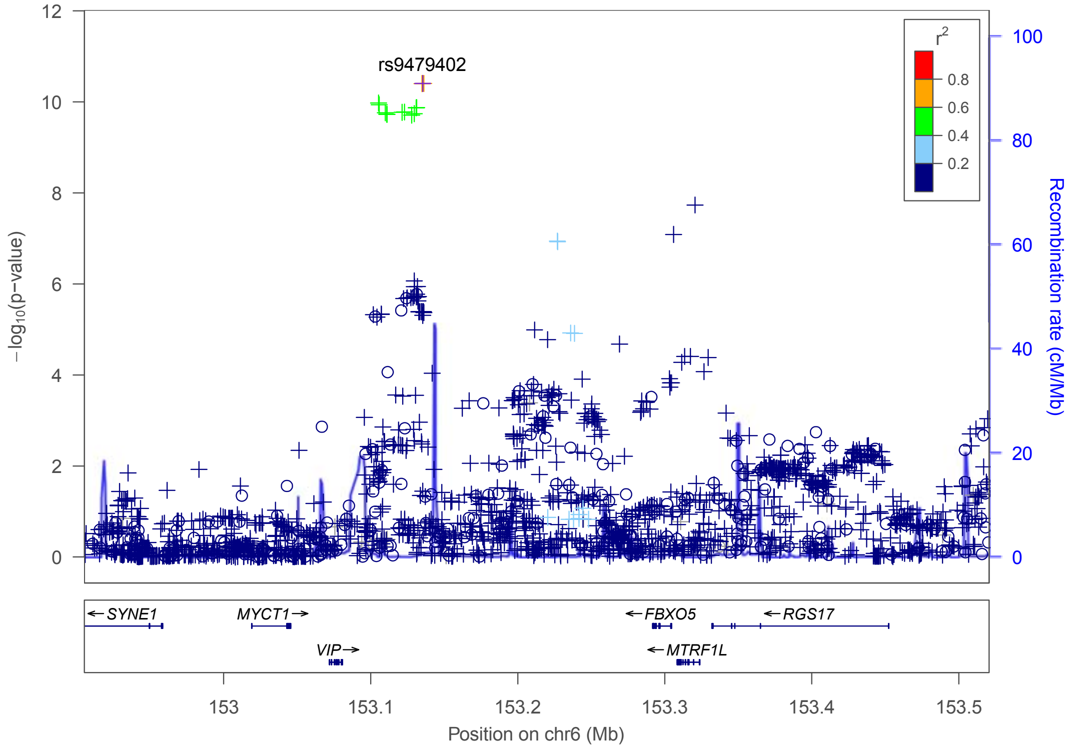

Figure S5. Regional plot for SNP rs55694368, close to gene *PER2*

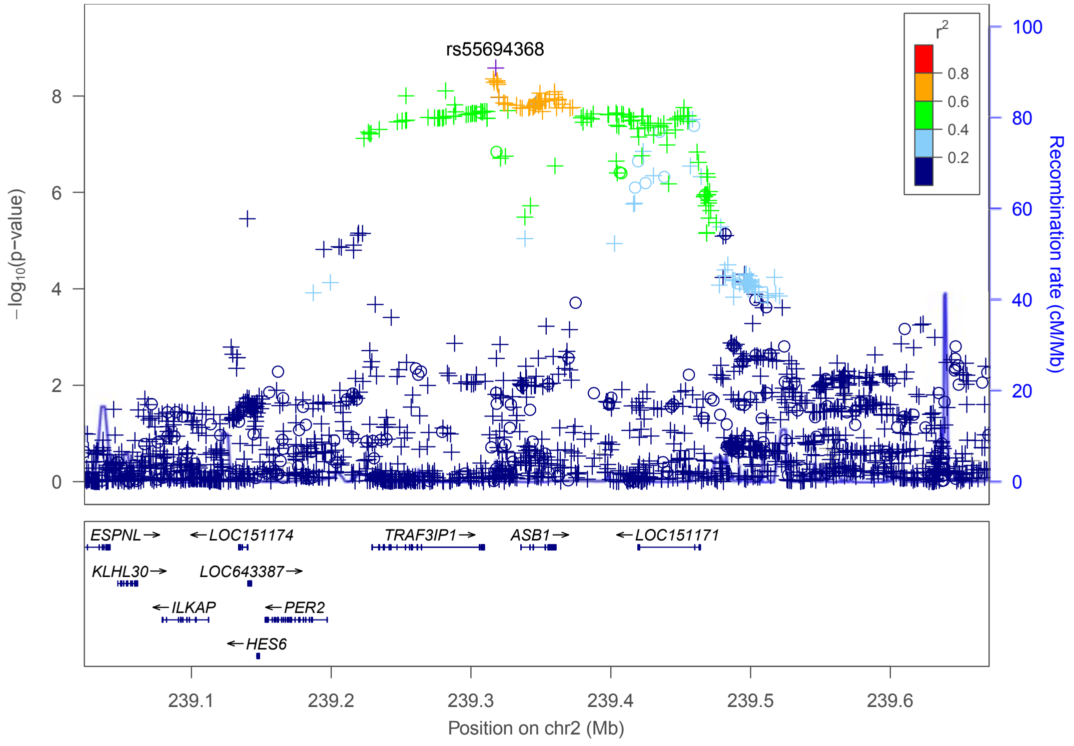

Figure S6. Regional plot for SNP rs35833281, close to gene *HCRTR2*

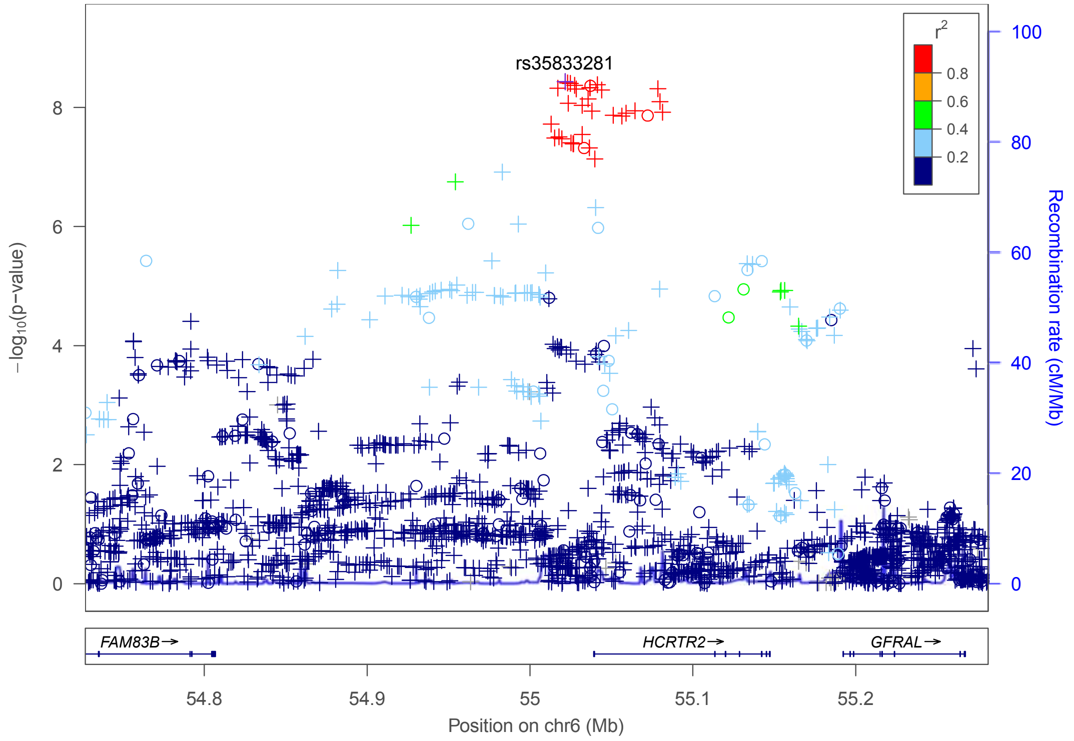

Figure S7. Regional plot for SNP rs11545787, close to *RASD1*

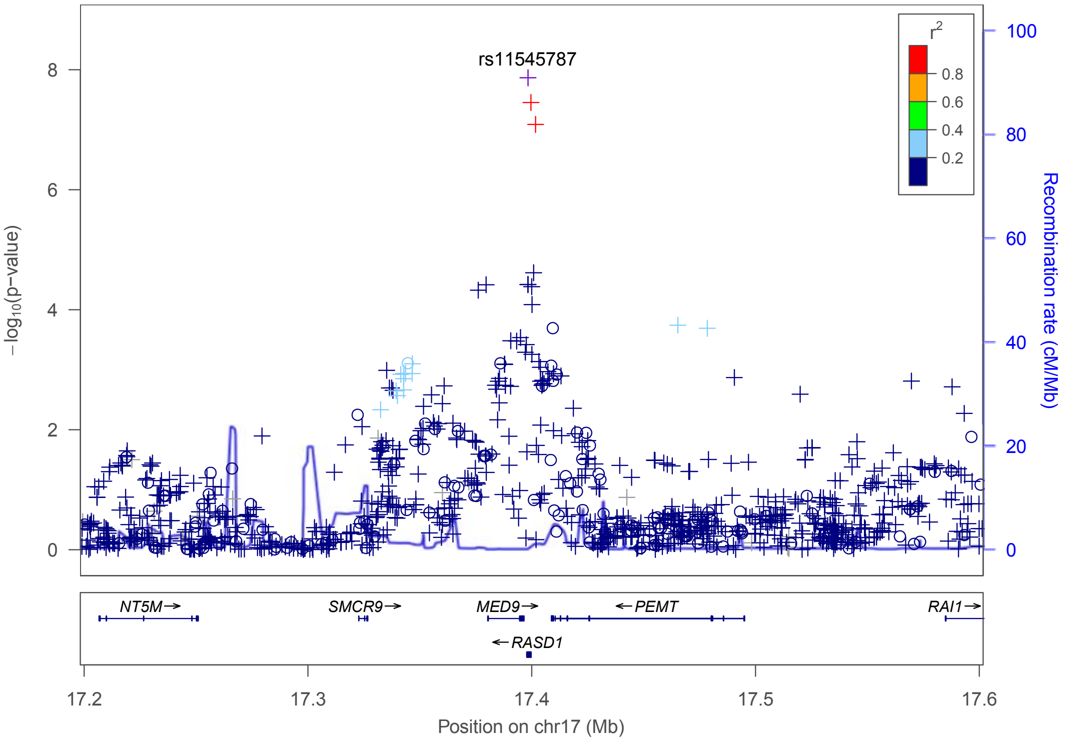

Figure S8. Regional plot for SNP rs11121022, close to *PER3*

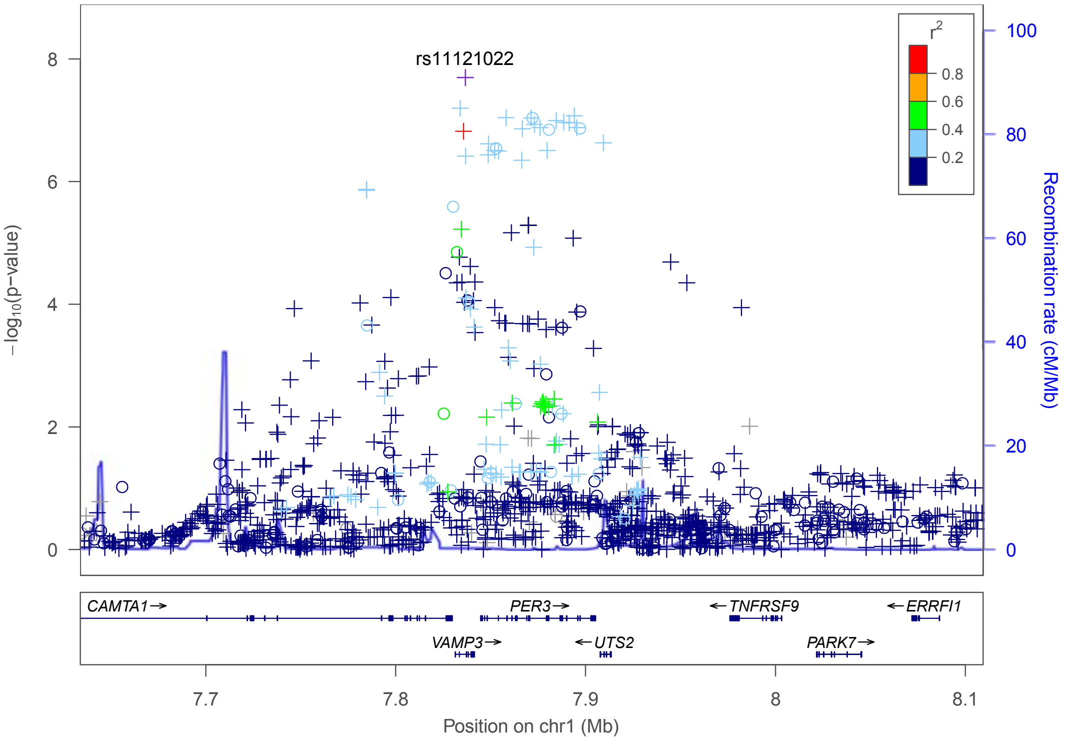

Figure S9. Regional plot for SNP rs9565309, close to *FBXL3*

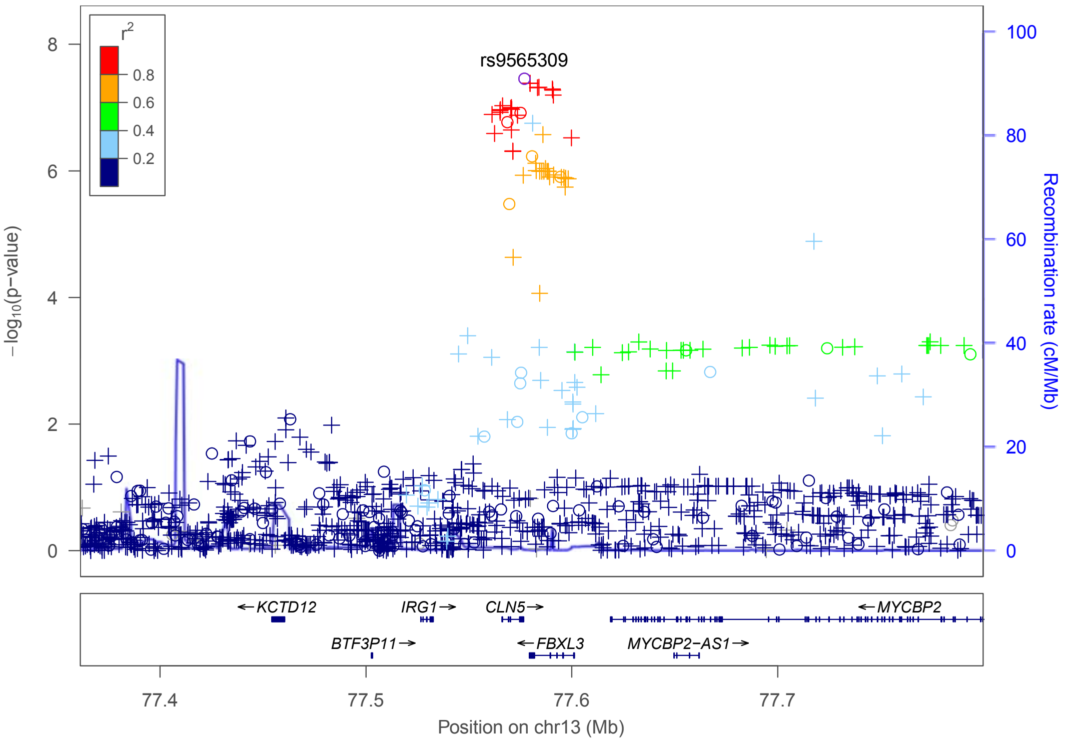

Figure S10. Regional plot for SNP rs1595824, close to *PLCL1*

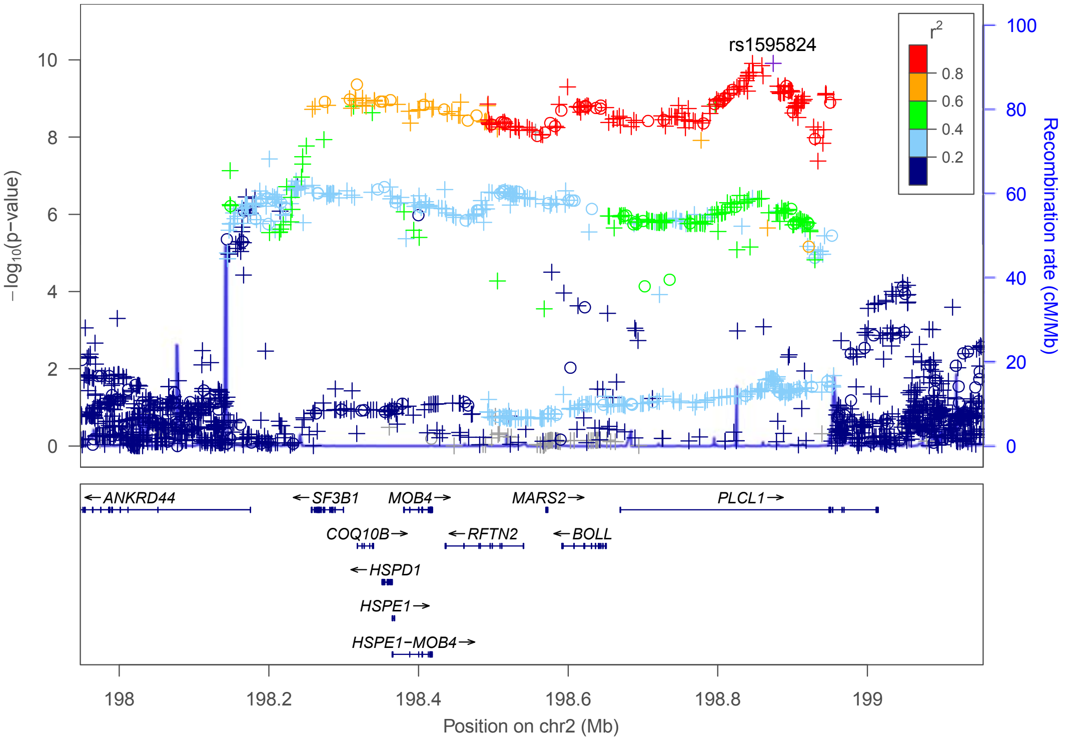

Figure S11. Regional plot for SNP rs34714364, close to *APH1A*

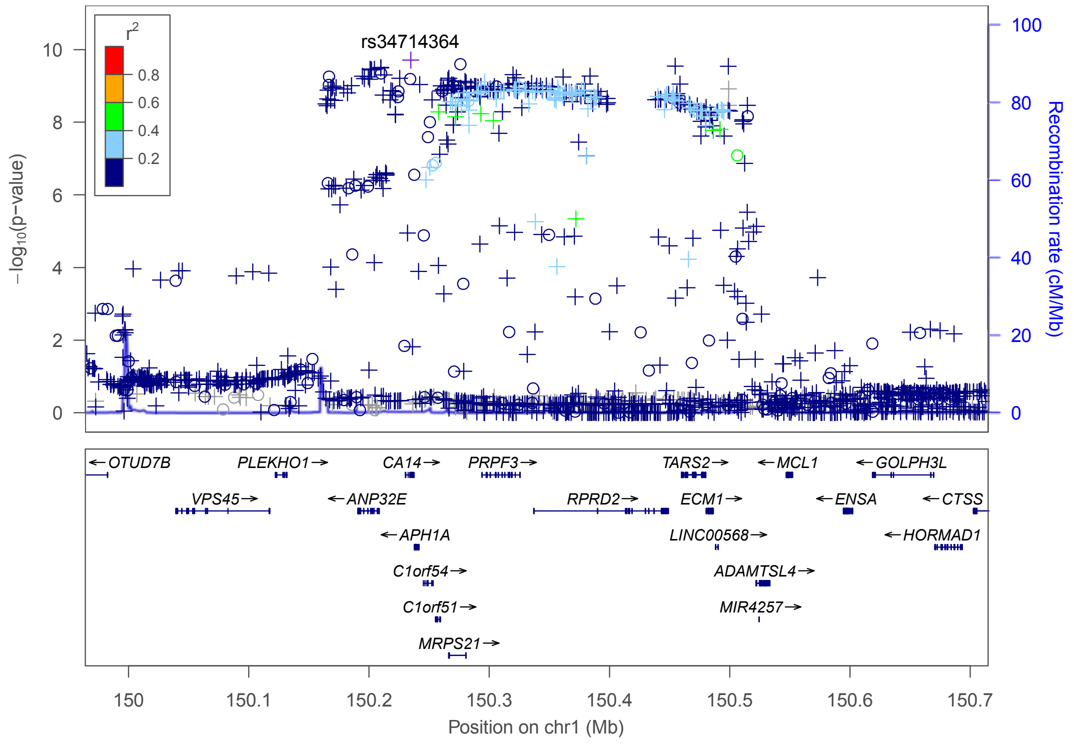

Figure S12. Regional plot for SNP rs3972456, close to *FBXL13*

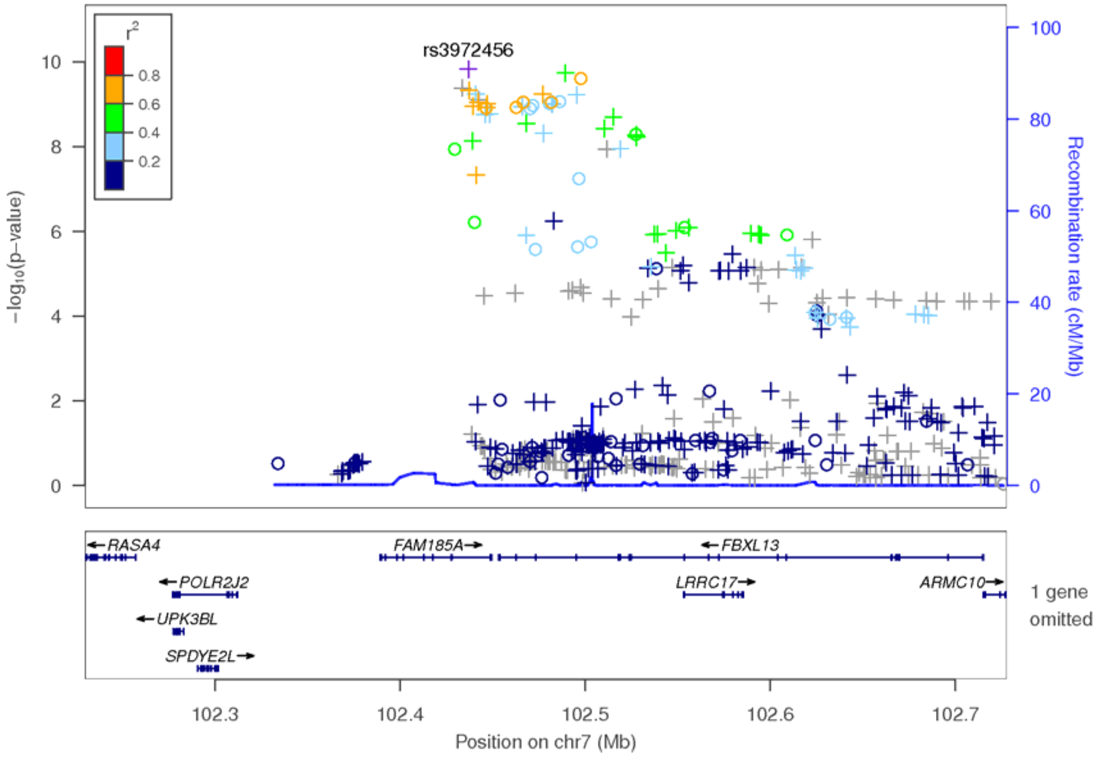

Figure S13. Regional plot for SNP rs12965577, close to *NOL4*

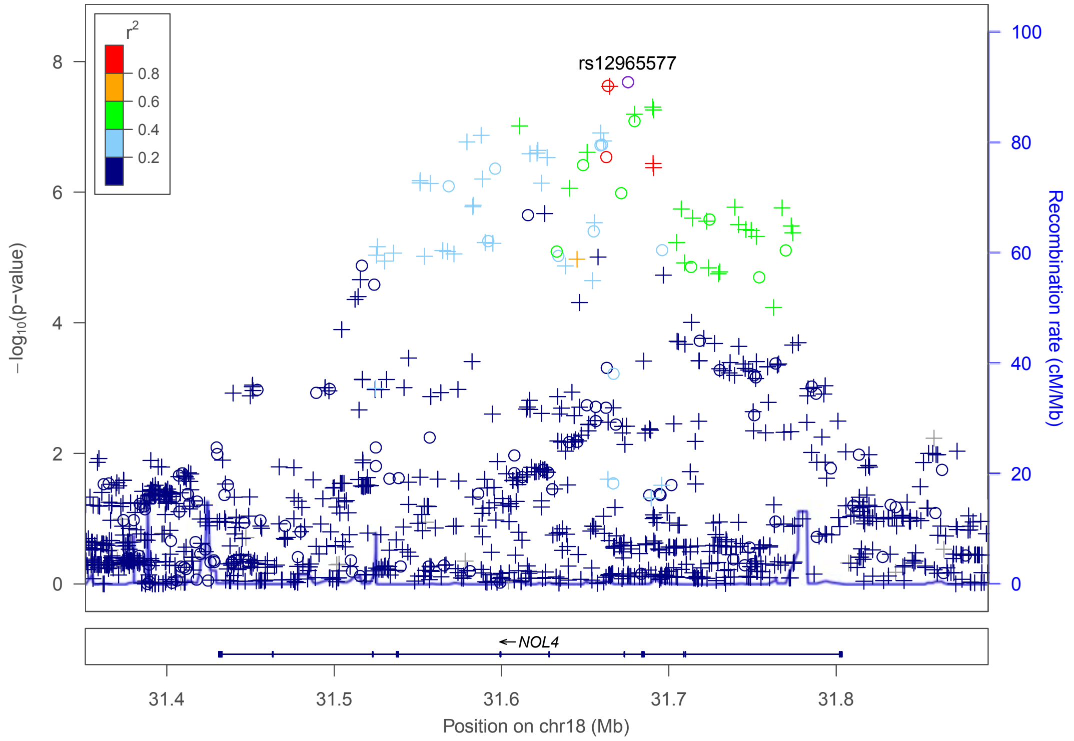

Figure S14. Regional plot for SNP rs12927162, close to *TOX3*

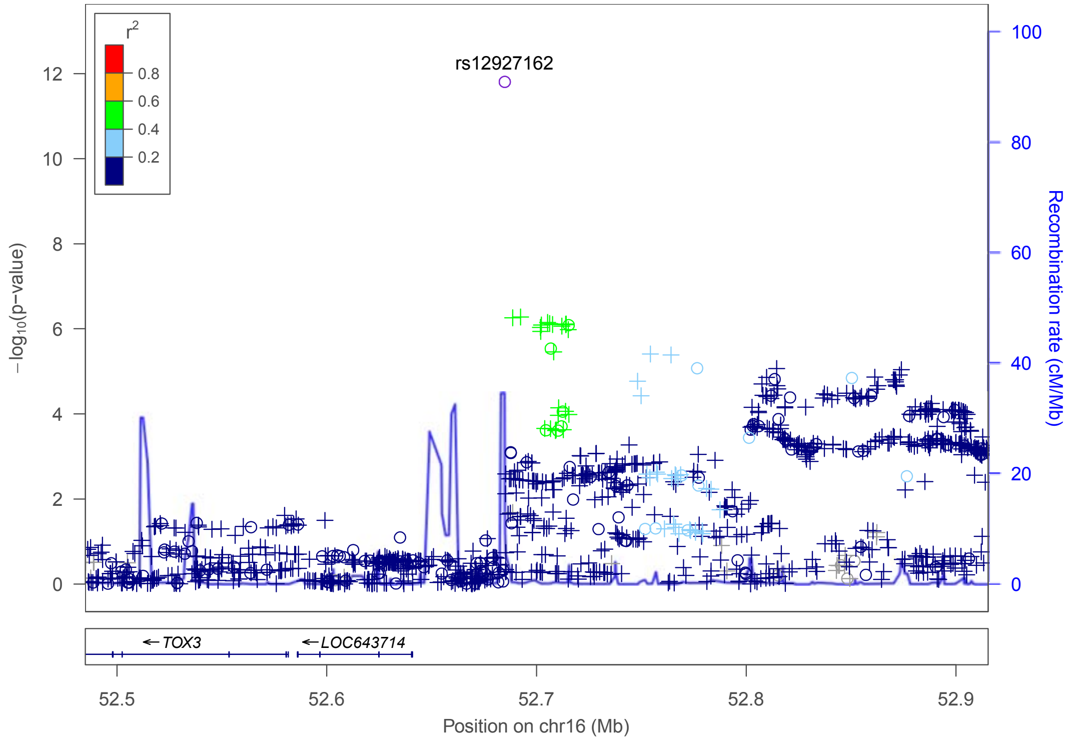

Figure S15. Regional plot for SNP rs10493596, close to *AK5*

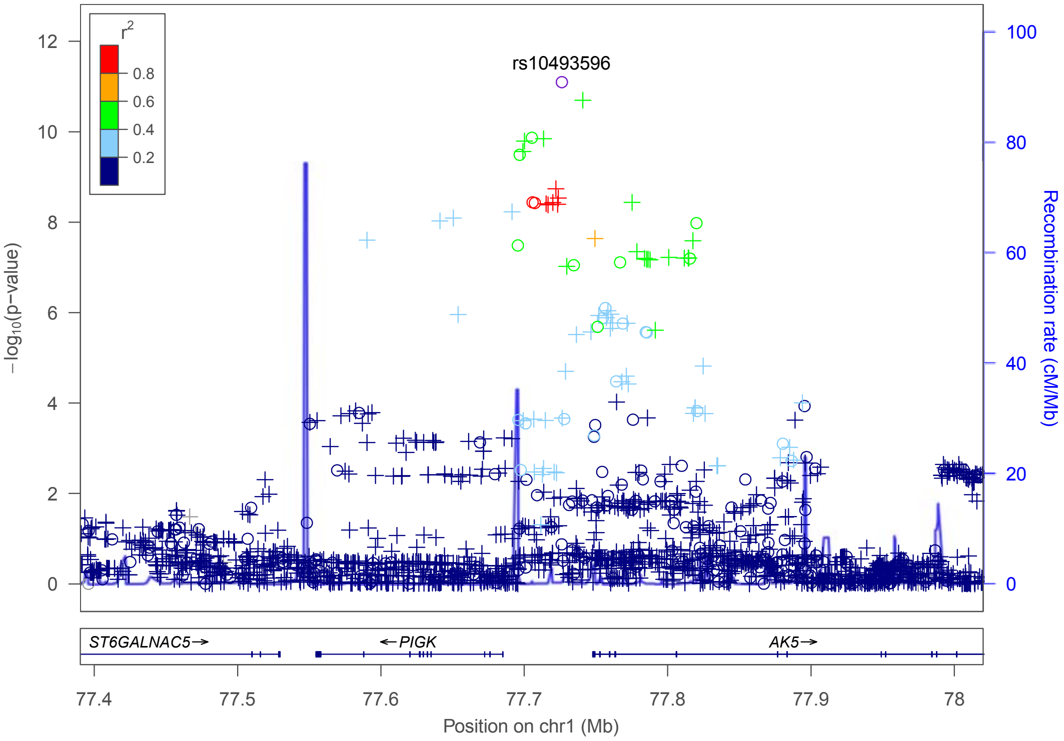

Figure S16. Regional plot for SNP rs2948276, close to *DLX5*

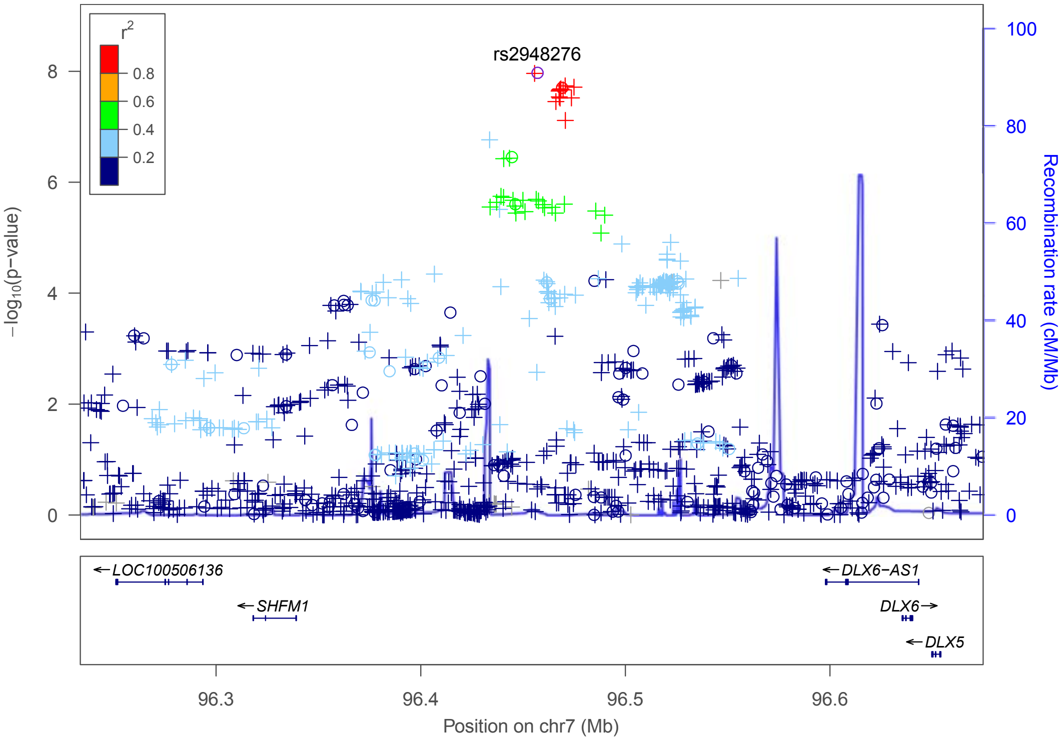

Figure S17. Regional plot for SNP rs6582618, close to *ALG10B*

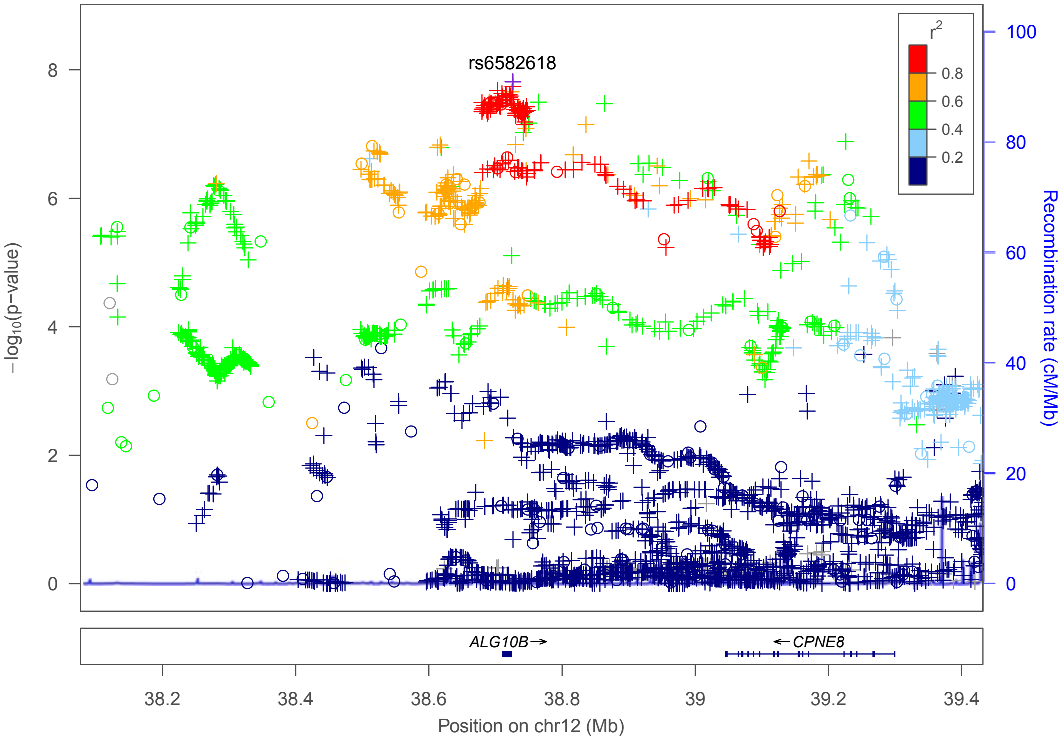

Figure S18. Comparisons of *chronotype* and *morning person* GWAS results. Loci colored in light red are genome-wide-significant ($P < 5\times10^{-8}$) in both analyses, and in dark red are significant in one analysis.

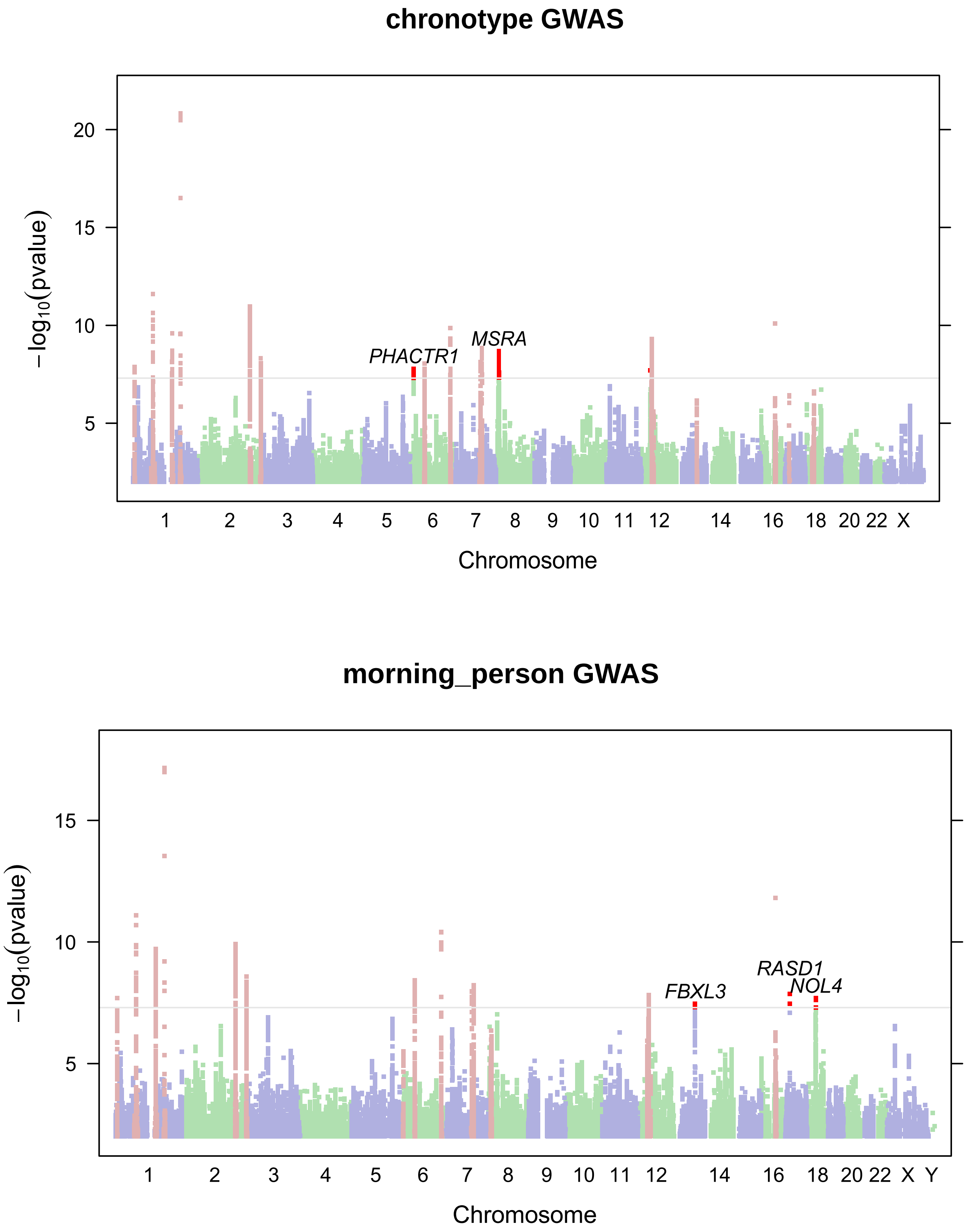

Figure S19-Figure S20 Regional association plots of *chronotype* GWAS specific significant loci

Figure S19. Region plot for rs2975734 in *MSRA*

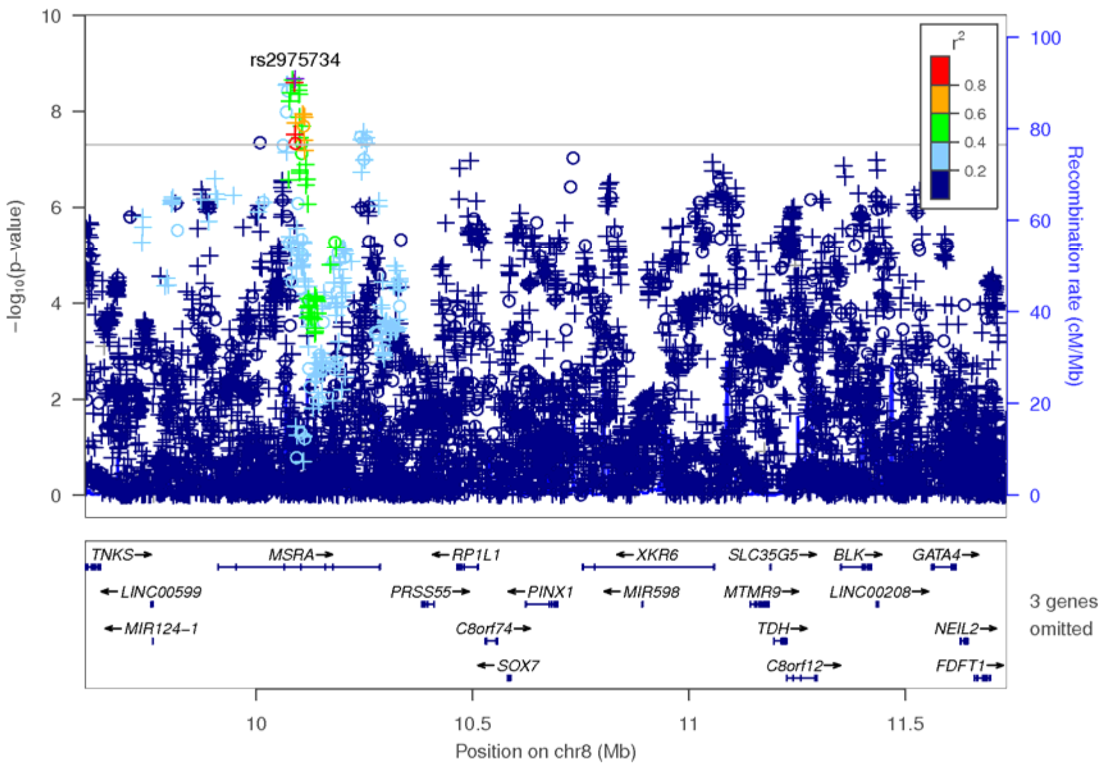

Figure S20. Region plot for rs9357620 in *PHACTR1*

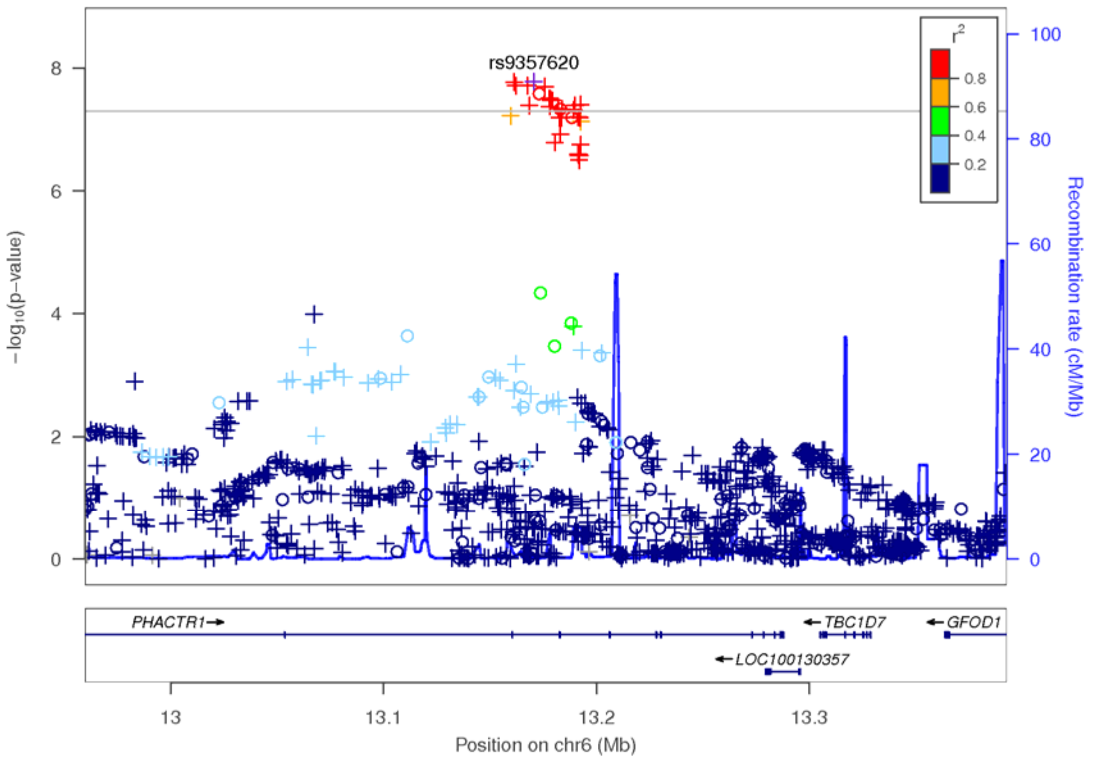

Supplementary Tables

Table S1. Summary of important circadian genes

Gene	Model organism	Reference	Note
PER	Drosophila	[50]	First discovery of circadian clock gene
CLOCK	Mice	[51]	First discovered mammalian clock gene
CRY	Drosophila	[52]	--
BMAL1	Mice	[53]	This gene is also known as ARNTL
NPAS	Mice	[54]	--
PER2	Human	[7,55]	Identified in a study of familial advanced sleep phase syndrome
CLOCK	Human	[56,57]	Candidate gene studies
PER3	Human	[21,58]	Candidate gene studies
BMAL1	Human	[59]	Candidate gene studies

Note: Also see review articles [8,60–62]

Table S2. Marginal associations of morning person versus sleep phenotypes, BMI and depression

A. Association of other binary phenotypes and morning person. The effect size describes the ratio of the odds of being a case in the other phenotype for a morning person than that for a night person.

Other phenotype	Sample size	Effect size (Morning vs. night persons)	95% CI	P value
**Insomnia**	64,049	0.66	[0.63, 0.69]	2.4×10^{-74}
**Sleep apnea**	60,184	0.94	[0.89, 0.98]	0.011
**Sleep needed (≥ 8 hours)**	32,114	0.66	[0.63, 0.69]	1.1×10^{-72}
**Sound sleeper**	41,755	0.74	[0.72, 0.77]	8.5×10^{-50}
**Restless leg syndrome**	30,954	0.91	[0.84, 0.995]	0.038
**Sweat while sleeping**	41,393	0.80	[0.77, 0.84]	1.0×10^{-23}
**Sleep walk**	31,533	0.76	[0.70, 0.83]	4.7×10^{-10}
**Average daily sleep duration (≥ 8 hours)**	28,245	0.96	[0.91, 1.0]	0.097
**Depression**	61,191	0.64	[0.61, 0.66]	1.1×10^{-128}

B. Association of continuous BMI and morning person. The effect size describes the difference of average BMI in kg/m^2 in morning persons than that in the night persons.

Other phenotype	Sample size	Effect size* (Morning vs. night persons)	95% CI	P value
**BMI**	80,042	-0.45	[-0.53, -0.37]	$1.5\text{x}10^{-28}$

Table S3. Definitions of other sleep phenotypes, BMI and depression

Phenotype	Related survey questions	Definition of phenotypes
**Morning person**	**S1**: Are you naturally a night person or a morning person?<br>(Night owl, Early bird, Neither)<br>**S2**: Are you naturally a night person or a morning person?<br>(Night person, Morning person, Neither, It depends, I'm not sure)	**Exclude neutral responses in S1 and S2 and then merge*** S1, S2
**Insomnia**	**S1**: Do you routinely have trouble getting to sleep at nights?<br>(Yes, No, I'm not sure)	**Case: Yes**<br>**Control: No**
**Sleep apnea**	**S1:** Have you ever been diagnosed by a doctor with any of the following neurological conditions?/"Sleep apnea" (interrupted or shallow breathing during sleep)<br>(Yes, No, I'm not sure)<br>**S2**: Have you ever been diagnosed with sleep apnea?<br>(Yes, No, I'm not sure)	**Merge** S1 – S2
**Sleep needed**	**S1**: How many hours of sleep do you feel like you need to function well during the day?<br>(Less than 4, 4-5, 6-7, 8-9, 10-11, More than 11, I'm not sure)	**Case**: (8-9, 10-11, more than 11)<br>**Control**: (less than 4, 4-5, 6-7)
**Sound sleeper**	**S1**: Are you a sound sleeper (generally able to sleep through noise and other disturbances)?<br>(Yes, No, I'm not sure)	**Case**: Yes<br>**Control**: No
**Restless leg syndrome**	**S1**: Have you ever been diagnosed with restless leg syndrome?<br>(Yes, No, I'm not sure)	**Case**: Yes<br>**Control**: No
**Sweat while sleeping**	**S1**: Even when sleeping at room temperature, do you sweat a noticeable amount while asleep?<br>(Yes, No, I'm not sure)	**Case**: Yes<br>**Control**: No
**Sleep walk**	**S1**: How often do you sleep walk?<br>(Never or almost never, Rarely, Sometimes, Often, Always or almost always, I'm not sure)	**Case**: (Sometimes, Often, Always or almost always)<br>**Control**: (Never or almost never, Rarely)
**Average daily sleep duration**	**S1**: How many hours of sleep do you get on a typical night?<br>(Less than 4, 4-5, 6-7, 8-9, 10-11, More than 11, I'm not sure)	**Case**: (8-9, 10-11, More than 11)<br>**Control**: (less than 4, 4-5, 6-7)
**Height** (inch)	**S1: "**Health habits": "Height (in)<br>**S2**: What is your height?<br>**S3**: Height (mm)?<br>**S4**: Height (ft and in)?<br>**S5**: Height (cm)?	**Transform** the height unit to inch and coalesce S1 – S5, **restrict** height to between 48 to 90 and **restrict** age to older than 18 years
**Weight** (lbs)	**S1**: "Health habits": "Weight (in lbs)?<br>**S2**: What is your current weight?<br>**S3**: What is your weight?<br>**S4**: Weight (in kgs)?<br>**S5**: Weight (in lbs)?	**Transform** the weight unit to be lbs and then **coalesce** S1 – S5, **restrict** BMI to between 5 and 70 and **restrict** age to older than 18 years.
**BMI**	Derived from height and weight.	$(\text{Weight}/2.2)/(\text{height}/39.37)^2$ and **restrict** BMI to between 5 and 70.
**Depression**	**S1**: Have you ever been diagnosed by a doctor with any of the following psychiatric conditions/"Depression" ? (Yes, No, I don't know)<br>**S2**: Have you ever been diagnosed with clinical depression? (Yes, No, I'm not sure)	**Merge** S1 – S2
**Alcohol abuse**	**S1:** Have you ever had any of the following type of substance abuse problems? (Alcoholism: Yes, No, I don't know)<br>**S2:** Have you ever drunk alcohol (at least one drink most weeks)? (Yes, No)<br>**S3:** Do you regularly drink alcohol (at least one drink most weeks)? (Yes, No)<br>**S4:** How often do you drink? (One drink = one can of beer, one glass of wine, one shot or one mixed drink?) (0-3/week, 4-6/week, 1-2/day, 3-6/day, 6+/day)<br>**S5:** Was there ever a 12 month period in your life in which you drank two or more drinks a day, even days a week? (Yes, No)	Cases reported having ever had alcoholism; for regular past or current drinkers, drinking 3 or more drinks per day; or ever having a 12-month period of 2 or more drinks a day. Controls were required to be age 35 or older and did not meet any of the criteria for cases.

**Nicotine abuse**	**S1:** Have you ever had any of the following types of substance abuse problems? (Nicotine abuse: Yes, No, I don't know)	Case: Yes, Control: No
**Current caffeine use**	**S1:** What types of caffeinated beverages do you drink regularly? Please check all that apply. (Coffee, tea, soda, energy drinks, other)<br>S1.1 On average, how many cups (~8 fluid ounces) of regular coffee do you drink a day? (<1, 1-2, 3-5, 6-8, 9-12, 13+)<br>S1.2 On average, how many cans (~8 fluid ounces) of caffeinated tea do you drink a day? (<1, 1-2, 3-5, 6-8, 9-12, 13+)<br>S1.3 On average, how many cans (~12 fluid ounces) of caffeinated soda do you drink a day? (<1, 1-2, 3-5, 6-8, 9-12, 13+)<br>S1.4 On average, how many cans/bottles (~8 fluid ounces) of caffeinated energy drink do you drink a day? (<1, 1-2, 3-5, 6-8, 9-12, 13+)	The responses are scored as 0.5, 1.5, 4.5, 7.0, 10 and 13 units per day. These are converted to mg/day using conversions of 150 (S1.1), 50 (S1.2), 35 (S1.3), 80 (S1.4). Individuals who do not report being regular drinkers are scored as 0 mg/day for the corresponding beverage types.

* The merge operator assigns a missing value when answers to surveys are both non-missing and are discordant. When one is non-missing and the other is missing, it will just use the non-missing value.
** The coalesce operator iterates through a list of survey questions and takes the first non-missing value out of the list of surveys.

Table S4. Association of 15 significant SNPs and other phenotypes

A. Association of 15 index SNPs using 23andme cohort.

Gene context: *RGS16*, Marker name: rs12736689

	**pvalue**	**effect**	**stderr**
Morning person	2.3e-21	-0.3	0.031
Insomnia	0.29	-0.044	0.042
Sleep apnea	0.55	-0.028	0.047
Sleep needed	0.28	-0.049	0.045
Sound sleeper	0.54	0.024	0.039
Restless leg syndrome	0.37	-0.072	0.08
Sweat while sleeping	0.3	-0.043	0.042
Sleep walk	0.55	0.048	0.082
Average daily sleep duration	0.35	-0.046	0.05
Depression	0.56	-0.019	0.032
BMI	0.95	0.0038	0.059

Gene context: *VIP*, Marker name: rs9479402

	**pvalue**	**effect**	**stderr**
Morning person	3.4e-13	-0.37	0.05
Insomnia	0.035	-0.15	0.07
Sleep apnea	0.24	0.092	0.079
Sleep needed	0.17	-0.1	0.072
Sound sleeper	0.086	0.11	0.063
Restless leg syndrome	0.51	0.092	0.14
Sweat while sleeping	0.42	0.056	0.07
Sleep walk	0.92	0.013	0.14
Average daily sleep duration	0.012	-0.2	0.079
Depression	0.019	0.13	0.054
BMI	0.036	0.2	0.094

Gene context: *PER2*, Marker name: rs55694368

	**pvalue**	**effect**	**stderr**
Morning person	5.5e-11	-0.16	0.024
Insomnia	0.28	0.034	0.031
Sleep apnea	0.6	0.019	0.036
Sleep needed	0.28	0.036	0.033
Sound sleeper	0.53	0.018	0.029
Restless leg syndrome	0.29	0.063	0.059
Sweat while sleeping	0.72	0.011	0.032
Sleep walk	0.16	0.084	0.059
Average daily sleep duration	0.92	-0.0039	0.037
Depression	0.83	0.0054	0.024
BMI	0.66	0.02	0.045

Gene context: *HCRTR2*, Marker name: rs35833281

	**pvalue**	**effect**	**stderr**
Morning person	8.3e-11	-0.081	0.012
Insomnia	0.12	-0.025	0.016
Sleep apnea	0.31	0.019	0.019
Sleep needed	0.66	0.0076	0.017
Sound sleeper	0.39	-0.013	0.015
Restless leg syndrome	0.59	-0.017	0.032
Sweat while sleeping	0.82	-0.0038	0.017
Sleep walk	0.074	-0.056	0.031
Average daily sleep duration	0.67	0.0083	0.019
Depression	0.32	0.013	0.013
BMI	0.18	-0.031	0.023

Gene context: *RASD1*, Marker name: rs11545787

	pvalue	effect	stderr
Morning person	4e-10	0.078	0.013
Insomnia	0.028	0.036	0.016
Sleep apnea	0.96	-0.00096	0.019
Sleep needed	0.94	-0.0013	0.018
Sound sleeper	0.54	-0.0093	0.015
Restless leg syndrome	0.27	0.036	0.032
Sweat while sleeping	0.16	0.024	0.017
Sleep walk	0.38	-0.028	0.032
Average daily sleep duration	0.76	0.0061	0.02
Depression	0.16	-0.018	0.013
BMI	0.042	-0.047	0.023

Gene context: *PER3*, Marker name: rs11121022

	pvalue	effect	stderr
Morning person	6.4e-10	0.064	0.01
Insomnia	0.65	-0.0063	0.014
Sleep apnea	0.87	0.0025	0.015
Sleep needed	0.13	0.022	0.014
Sound sleeper	0.004	-0.036	0.013
Restless leg syndrome	0.13	0.04	0.026
Sweat while sleeping	0.86	-0.0025	0.014
Sleep walk	0.088	0.045	0.026
Average daily sleep duration	0.47	0.012	0.016
Depression	0.14	-0.016	0.011
BMI	0.89	-0.0027	0.019

Gene context: *FBXL3*, Marker name: rs9565309

	pvalue	effect	stderr
Morning person	1.2e-09	0.17	0.029
Insomnia	0.026	0.082	0.037
Sleep apnea	0.76	0.013	0.042
Sleep needed	0.92	0.0042	0.039
Sound sleeper	0.64	-0.016	0.034
Restless leg syndrome	0.23	-0.085	0.07
Sweat while sleeping	0.85	0.007	0.038
Sleep walk	0.16	-0.099	0.069
Average daily sleep duration	0.83	0.0096	0.045
Depression	0.44	0.022	0.029
BMI	0.49	0.036	0.052

Gene context: *PLCL1*, Marker name: rs1595824

	pvalue	effect	stderr
Morning person	1.4e-12	0.072	0.01
Insomnia	0.02	0.031	0.013
Sleep apnea	0.061	0.028	0.015
Sleep needed	0.35	0.013	0.014
Sound sleeper	0.42	0.01	0.012
Restless leg syndrome	1	0.0001	0.026
Sweat while sleeping	0.62	0.0066	0.014
Sleep walk	0.21	-0.032	0.026
Average daily sleep duration	0.5	0.011	0.016
Depression	0.1	-0.017	0.01
BMI	0.14	0.028	0.019

Gene context: *APH1A*, Marker name: rs34714364

	pvalue	effect	stderr
Morning person	2.4e-12	0.12	0.017
Insomnia	0.17	-0.03	0.018

Sleep apnea	0.37	-0.022	0.025
Sleep needed	0.66	0.01	0.023
Sound sleeper	0.75	-0.0064	0.02
Restless leg syndrome	0.98	-0.0011	0.043
Sweat while sleeping	0.33	0.021	0.022
Sleep walk	0.95	0.0027	0.042
Average daily sleep duration	0.89	-0.0038	0.026
Depression	0.53	0.011	0.017
BMI	0.78	0.0084	0.031

Gene context: *FBXL13*, Marker name: rs3972456

	**pvalue**	**effect**	**stderr**
Morning person	1.5e-10	-0.087	0.014
Insomnia	0.054	-0.035	0.018
Sleep apnea	0.5	-0.014	0.02
Sleep needed	0.33	-0.019	0.019
Sound sleeper	0.47	-0.012	0.017
Restless leg syndrome	0.21	-0.044	0.035
Sweat while sleeping	0.59	0.0097	0.018
Sleep walk	0.57	-0.02	0.035
Average daily sleep duration	0.56	-0.012	0.021
Depression	0.14	0.021	0.014
BMI	0.61	0.013	0.025

Gene context: *NOL4*, Marker name: rs12965577

	**pvalue**	**effect**	**stderr**
Morning person	6.6e-10	-0.066	0.011
Insomnia	0.94	-0.001	0.014
Sleep apnea	0.82	0.0037	0.016
Sleep needed	0.78	0.0042	0.015
Sound sleeper	0.76	0.004	0.013
Restless leg syndrome	0.069	0.05	0.027
Sweat while sleeping	0.4	-0.012	0.014
Sleep walk	0.069	0.049	0.027
Average daily sleep duration	0.53	-0.011	0.017
Depression	0.083	0.019	0.011
BMI	0.046	0.04	0.02

Gene context: *TOX3*, Marker name: rs12927162

	**pvalue**	**effect**	**stderr**
Morning person	6.9e-15	-0.091	0.012
Insomnia	0.01	0.039	0.015
Sleep apnea	0.5	-0.012	0.018
Sleep needed	0.18	0.022	0.016
Sound sleeper	0.46	-0.01	0.014
Restless leg syndrome	0.56	0.017	0.03
Sweat while sleeping	0.34	-0.015	0.016
Sleep walk	0.45	0.022	0.029
Average daily sleep duration	0.042	-0.037	0.018
Depression	0.66	0.0052	0.012
BMI	0.99	-0.00014	0.022

Gene context: *AK5*, Marker name: rs10493596

	**pvalue**	**effect**	**stderr**
Morning person	5e-14	0.089	0.012
Insomnia	0.42	-0.013	0.016
Sleep apnea	0.67	-0.0076	0.018
Sleep needed	0.62	-0.0083	0.017
Sound sleeper	0.23	0.018	0.015

Restless leg syndrome	0.83	0.0065	0.031
Sweat while sleeping	0.52	0.01	0.016
Sleep walk	0.42	0.025	0.03
Average daily sleep duration	0.42	0.015	0.019
Depression	0.42	-0.0099	0.012
BMI	0.51	-0.015	0.022

Gene context: *DLX5*, Marker name: rs2948276

	pvalue	effect	stderr
Morning person	3e-10	-0.084	0.013
Insomnia	0.038	0.037	0.018
Sleep apnea	0.84	0.004	0.02
Sleep needed	0.91	0.0022	0.019
Sound sleeper	0.41	-0.013	0.016
Restless leg syndrome	0.59	0.019	0.034
Sweat while sleeping	0.87	-0.0028	0.018
Sleep walk	0.4	0.028	0.034
Average daily sleep duration	0.47	-0.015	0.021
Depression	0.29	0.014	0.014
BMI	0.31	0.025	0.025

Gene context: *ALG10B*, Marker name: rs6582618

	pvalue	effect	stderr
Morning person	4.6e-10	0.066	0.011
Insomnia	0.35	0.013	0.013
Sleep apnea	0.018	0.037	0.016
Sleep needed	0.63	0.0071	0.015
Sound sleeper	0.65	-0.0059	0.013
Restless leg syndrome	0.39	0.024	0.027
Sweat while sleeping	0.63	0.0069	0.014
Sleep walk	0.24	0.032	0.027
Average daily sleep duration	0.49	-0.011	0.017
Depression	0.61	0.0056	0.011
BMI	0.043	0.04	0.02

B. Association of 15 SNPs or their proxies in the GIANT BMI GWAS

our.hits	distance (bp)	r^2	assay.name	effect direction for B allele	pvalue
rs12736689	0	1.000	rs12736689	-	0.092
rs9479402	-25450	0.775	rs9478358	+	0.595
rs55694368	138551	0.270	rs6749508	+	0.406
rs35833281	-83587	0.263	rs9382445	-	0.035
rs11545787	-53625	0.337	rs925095	+	0.770
rs11121022	16260	0.262	rs875994	-	0.019
rs9565309	0	1.000	rs9565309	+	0.812
rs1595824	0	1.000	rs1595824	-	0.180
rs34714364	86829	0.291	rs1625468	+	0.092
rs3972456	29834	0.657	rs1541519	+	0.099
rs12965577	0	1.000	rs12965577	-	0.282
rs12927162	0	1.000	rs12927162	-	0.067
rs10493596	0	1.000	rs10493596	-	0.699
rs2948276	0	1.000	rs2948276	-	0.391
rs6582618	-36200	0.874	rs4550281	-	0.028

We looked up our index SNP in the GIANT consortium published GWAS dataset. For those that do not have a SNP with matching name, we searched all SNPs that are within a 1Mb window of our index SNP and have a r^2 greater than 0.25. We used the SNP with the smallest P value as the proxy SNP. Since we used the original BMI instead of the scaled BMI in the GIANT analysis, the effect size is not directly comparable. We instead reported the sign of the effect size in the table.

C. Association of 15 SNPs or their proxies in the CONVERGE major depressive disorder GWAS

our.hits	distance (bp)	r^2	assay.name	Odds ratio for B allele	pvalue
rs12736689	14575	0.539	rs1148776	0.985	0.344
rs9479402	0	1.000	rs9479402	0.997	0.667
rs55694368	0	1.000	rs55694368	1.012	0.453
rs35833281	0	1.000	rs35833281	0.999	0.958
rs11545787	0	1.000	rs11545787	0.988	0.327
rs11121022	0	1.000	rs11121022	1.000	0.957
rs9565309	0	1.000	rs9565309	0.991	0.344
rs1595824	0	1.000	rs1595824	1.001	0.880
rs34714364	48253	0.296	rs522435	0.991	0.279
rs3972456	294874	0.296	rs4729873	0.981	0.007
rs12965577	0	1.000	rs12965577	1.004	0.568
rs12927162	3790	0.427	rs4784233	1.003	0.652
rs10493596	0	1.000	rs10493596	1.017	0.040
rs2948276	0	1.000	rs2948276	1.001	0.917
rs6582618	0	1.000	rs6582618	1.010	0.261

We looked up our index SNP in the CONVERGE consortium published major depressive disorder GWAS dataset. For those that do not have a SNP with matching name, we searched all SNPs that are within a 1Mb window of our index SNP and have a r^2 greater than 0.25. We used the SNP with the smallest P value as the proxy SNP.

Table S5. SNPs used for the calculation of BMI genetic risk and their association with morningness

Nearest gene	SNP[1]	A allele	B allele	Beta[2] (Effect of B allele)	P value[3] for association with morningness
FTO	rs1558902	T	A	0.39	6.0×10^{-6}
TMEM18	rs2867125	C	T	-0.31	0.86
MC4R	rs571312	C	A	0.23	0.18
GNPDA2	rs10938397	A	G	0.18	0.87
BDNF	rs10767664	A	T	-0.19	0.02
NEGR1	rs2815752	A	G	-0.13	0.85
SH2B1	rs7359397	C	T	0.15	0.95
ETV5	rs9816226	T	A	-0.14	0.8
MTCH2	rs3817334	C	T	0.06	1.1×10^{-4}
KCTD15	rs29941	G	A	-0.06	0.09
RBJ	rs713586	T	C	0.14	0.04
GPRC5B	rs12444979	C	T	-0.17	0.12
MAP2K5	rs2241423	G	A	-0.13	0.13
QPCTL	rs2287019	C	T	-0.15	0.75
TNNI3K	rs1514175	G	A	0.07	0.35
SLC39A8	rs13107325	C	T	0.19	0.05
FLJ35779	rs2112347	T	G	-0.10	5.8×10^{-3}
LRRN6C	rs10968576	A	G	0.11	0.35
TMEM160	rs3810291	A	G	-0.09	0.37
FANCL	rs887912	C	T	0.10	0.06
CADM2	rs13078807	A	G	0.10	0.55
PRKD1	rs11847697	C	T	0.17	0.63
LRP1B	rs2890652	T	C	0.09	0.81
PTBP2	rs1555543	C	A	-0.06	0.30
MTIF3	rs4771122	A	G	0.09	0.24
ZNF608	rs4836133	A	C	-0.07	0.47
RPL27A	rs4929949	T	C	0.06	0.56
NUDT3	rs206936	A	G	0.06	0.55

1. This list of SNPs that are associated with BMI is from Table 1 in [39].
2. Beta is the change of BMI (in kg/m^2) per additional copy of the B alleles.
3. P value is calculated by regressing morningness against the SNP, adjusting for age, sex and five PCs.

Table S6. Summary of joint model results for conditional analysis

Gene context	Marker	Chromosome	Position	Alleles	Effect*	Stderr	P value
VIP	rs9479402	6	153135339	C/T	-0.331	0.051	$3.2x10^{-9}$
	rs62436127	6	153320618	C/G	-0.076	0.014	$1.6x10^{-6}$
APH1A	rs34714364	1	150234657	G/T	0.090	0.017	$2.8x10^{-6}$
	rs10888576	1	150200792	C/G	0.062	0.012	$5.0x10^{-6}$
PER2	rs55694368	2	239317692	G/T	-0.153	0.024	$7.1x10^{-9}$
	rs114769095	2	239140025	C/G	-0.205	0.042	$9.7x10^{-6}$

* Estimate is the log odds ratio from the logistic regression model of morning person versus age, sex, top 5 PCs and the genotype for the SNP.

Table S7. Detailed results of interaction analysis between significant SNPs and covariates including age, sex, BMI, alcohol abuse, nicotine abuse and current caffeine use

A. Main effect of each additional covariate

1. Continuous BMI

	Sample size	Estimate	Stderr	Z	P value
bmi	83807	-0.033	0.0014	-24	1e-125

2. Binary covariates

	Sample size	Estimate	Stderr	Z	P value
alcohol_abuse	40760	-0.092	0.029	-3.1	0.0018
nicotine_abuse	50893	-0.34	0.025	-13	3.9e-41
current_caffeine_use	30019	0.0001	4.9e-05	2.1	0.036

Regression results for the null model (a model without the SNP genotype term) of morningness versus age, sex, top 5 PCs and an additional covariate given by the row names such as BMI and alcohol abuse. The estimate is the log odds ratio.

B. Comparison of the main effect (in log odds ratio) of each SNP for the model with and without an additional covariate

1. Effect size for each SNP

SNP	original model	Additional covariate added to the original GWAS model				
		bmi	alcohol_abuse	nicotine_abuse	current_caffeine_use	all together
rs12736689	0.3	0.33	0.35	0.34	0.33	0.38
rs9479402	0.37	0.34	0.35	0.38	0.24	0.21
rs55694368	-0.16	-0.16	-0.19	-0.18	-0.21	-0.23
rs35833281	0.081	0.088	0.057	0.07	0.077	0.075
rs11545787	-0.078	-0.083	-0.061	-0.07	-0.059	-0.05
rs11121022	0.064	0.065	0.07	0.054	0.066	0.078
rs9565309	-0.17	-0.17	-0.16	-0.14	-0.15	-0.16
rs1595824	-0.072	-0.074	-0.09	-0.097	-0.095	-0.092
rs34714364	0.12	0.13	0.084	0.083	0.1	0.1
rs3972456	0.087	0.092	0.087	0.088	0.1	0.085
rs12965577	-0.066	-0.06	-0.065	-0.07	-0.082	-0.068
rs12927162	-0.091	-0.09	-0.082	-0.071	-0.093	-0.094
rs10493596	0.089	0.093	0.1	0.099	0.1	0.13
rs2948276	-0.084	-0.083	-0.074	-0.08	-0.071	-0.062
rs6582618	-0.066	-0.067	-0.084	-0.069	-0.079	-0.11

The original model column describes the effect size of each SNP in the original GWAS model of morningness with an adjustment of age, sex and top 5 PCs. Each cell in other columns is the effect size for the regression model of morningness versus SNP genotype, age, sex, top 5 PCs and each additional confounder given by the column name such as BMI and alcohol abuse. The "all together" column describes the effect size of each SNP in the model of morningness versus SNP genotype, age, sex top 5 PCs and all additional confounders including BMI, alcohol abuse, nicotine abuse and current caffeine use.

2. P value for each SNP

SNP	original model	Additional covariate added to the original GWAS model				
		bmi	alcohol_abuse	nicotine_abuse	current_caffeine_use	all together
rs12736689	2.2e-21	7.8e-24	4.2e-14	1.6e-16	1.4e-09	8.2e-10
rs9479402	7.5e-15	1.9e-13	1.2e-06	4.6e-06	4.7e-06	0.042
rs55694368	4.8e-14	7.7e-14	7.3e-09	3.6e-10	3.9e-07	8.2e-07
rs35833281	3.2e-13	1.4e-10	3.8e-06	4.4e-08	0.0083	0.0019
rs11545787	1.4e-12	4.5e-12	1.3e-09	9.7e-13	6.7e-08	0.041
rs11121022	2.3e-12	2.8e-14	0.00062	0.00019	0.00046	9.7e-05
rs9565309	6.1e-11	1.2e-10	3.1e-08	9.4e-09	5.6e-07	0.003
rs1595824	8.1e-11	1.5e-11	0.0017	2.6e-05	0.00034	3.3e-06

rs34714364	1.5e-10	1.3e-10	1.4e-05	1.2e-06	3.4e-05	0.0016
rs3972456	3.1e-10	3.7e-09	0.00014	7.5e-06	0.0024	0.0016
rs12965577	4.2e-10	3.8e-10	0.00082	3.5e-05	0.0068	0.0011
rs12927162	4.7e-10	1.5e-09	4.8e-08	1.1e-06	1.6e-05	3.3e-05
rs10493596	6.4e-10	1.7e-09	3.5e-06	9.8e-05	0.00023	7.5e-09
rs2948276	6.8e-10	8.2e-08	2.7e-05	1.1e-06	1.2e-05	0.019
rs6582618	1.5e-09	3.2e-08	7.6e-05	0.00023	0.0021	1.8e-07

The original model column is the p value of each SNP for the original GWAS model before the adjustment of genomic factor. Each cell in all other columns is the P value for the regression model of morningness versus genotype, age, sex, top 5 PCs and each additional covariate given by the column names such as BMI and alcohol abuse. The "all together" column describe the P value for each SNP in the model of morningness versus SNP genotype, age, sex top 5 PCs and all additional confounders including BMI, alcohol abuse, nicotine abuse and current caffeine use.

C. Testing for interaction effect between significant SNP and each confounder

**SNP**	**Covariate to test interaction with SNP genotypes**						
	**age**	**sex**	**bmi**	**alcohol_abuse**	**nicotine_abuse**	**current_caffeine_use**	**all together**
rs12736689	0.75	0.18	0.49	0.25	0.11	0.96	0.92
rs9479402	0.21	0.058	0.81	0.52	0.9	0.31	0.6
rs55694368	0.61	0.02	0.54	0.38	0.4	0.7	0.92
rs35833281	0.0046	0.086	0.47	0.79	0.15	0.54	0.73
rs11545787	0.053	0.79	0.061	0.066	0.095	0.47	0.046
rs11121022	0.51	0.82	0.034	0.082	0.056	0.057	0.021
rs9565309	0.33	0.86	0.02	0.99	0.38	0.0089	0.059
rs1595824	0.18	0.12	0.57	0.35	0.16	0.56	0.012
rs34714364	0.51	0.91	0.48	0.88	0.44	0.04	0.21
rs3972456	0.96	0.76	0.18	0.71	0.73	0.96	0.36
rs12965577	0.14	0.14	0.26	0.86	0.12	0.49	0.69
rs12927162	0.55	0.15	0.29	0.2	0.78	0.67	0.43
rs10493596	0.78	0.28	0.13	0.54	0.42	0.19	0.56
rs2948276	0.3	0.47	0.07	0.22	0.29	0.47	0.14
rs6582618	0.17	0.18	0.14	0.99	0.53	0.99	0.11

Each cell is the p value for the interaction term in the regression model of morningness versus age, sex, top 5 PCs, the genotype of each SNP, an additional confounder if the confounder is not age or sex, and the interaction term of SNP and confounder. The "all together" column describes the P value for the joint test of the interaction term of each SNP and all confounders, including age, sex, BMI, alcohol abuse, nicotine abuse and current caffeine use.

D. SNP effects (in log odds ratio) in different age groups

	Age < 45 (n = 49,282)		Age: 45 – 60 (n = 26,039)		Age >60 (n = 24,935)		Overall P value
	Estimate	P value	Estimate	P value	Estimate	P value	
rs12736689	0.31	7.3e-11	0.26	7.6e-06	0.28	6.8e-06	0.8
rs9479402	0.35	1.4e-06	0.25	0.0088	0.5	1e-06	0.3
rs55694368	-0.13	0.00057	-0.18	6.6e-05	-0.19	1.9e-05	0.77
rs35833281	0.12	6.8e-11	0.065	0.0047	0.035	0.13	0.015
rs11545787	-0.093	2.1e-06	-0.092	7.3e-05	-0.032	0.16	0.16
rs11121022	0.067	2.1e-05	0.098	2.7e-07	0.022	0.26	0.15
rs9565309	-0.12	0.0085	-0.27	4.9e-07	-0.15	0.0041	0.015
rs1595824	-0.08	3.1e-07	-0.09	1.8e-06	-0.049	0.01	0.21
rs34714364	0.11	7.2e-06	0.09	0.0036	0.15	2.9e-06	0.77
rs3972456	0.077	0.0002	0.11	5.3e-06	0.069	0.0072	0.22
rs12965577	-0.075	5e-06	-0.089	6.9e-06	-0.03	0.14	0.11
rs12927162	-0.11	1.6e-09	-0.062	0.0038	-0.086	8.2e-05	0.11
rs10493596	0.094	2.4e-07	0.076	0.00052	0.096	2e-05	0.57
rs2948276	-0.079	0.00014	-0.073	0.003	-0.099	6.7e-05	0.9
rs6582618	-0.041	0.011	-0.091	3.3e-06	-0.075	0.00014	0.12

We performed logistic regression of morningness versus the genotypes of each SNP, sex and top 5 PCs in samples from different age groups. Estimates are log odds ratios. The overall P value is to test whether the effects are the same across the three age groups.

Table S8. Power calculation for Mendelian randomization analysis

**Causality direction**	**Morningness→ Depression**		**Morningness→ BMI**		**BMI→ Morningness**	
Sample size	61,023		79,829		83,579	
Observed effect estimate	-0.50		-0.18		-0.19	
Simulations	**Effect***	**Power**	**Effect****	**Power**	**Effect*****	**Power**
	0	0.051	0	0.049	0	0.048
	-0.10	0.065	-0.10	0.134	-0.10	0.299
	-0.18	0.099	-0.20	0.371	-0.18	0.779
	-0.26	0.152	-0.30	0.700	-0.26	0.976
	-0.34	0.198	-0.40	0.923	-0.34	0.998
	-0.41	0.273	-0.50	0.988	-0.41	0.999
	-0.47	0.335	-0.60	1.0	-0.47	1.0
	-0.53	0.396	-0.70	1.0	-0.53	1.0
	-0.59	0.449				
	-0.64	0.509				
	-0.69	0.554				
	-0.91	0.757				
	-1.10	0.875				

Causality direction means which causal direction we want to make inference of. For example, for the case of "morningness→depression", we use the genetic risk of morningness as an instrument variable and test if morningness causes depression. Sample size is the number of samples where we have complete data for genetic risks, exposure, covariates and outcome variables. Observed effect is the effect size estimate from the regression of outcome versus age, sex, top 5 PCs and weighted genetic risk. In our simulations, we vary the transferred genetic effect size to simulate outcome, then run regression of outcome versus genetic risk, age, sex and top 5 PCs. We count the number of simulations when we see a P value for the genetic risk being less than 0.05. When the effect is 0, the power should be equal to the type I error rate (0.05)

* This effect is the transferred genetic effect, which describes the change of probability of depression per unit increase of probability of being a morning person.

**This effect is the transferred genetic effect, which describes the change of BMI in unit of kg m^{-2} per unit increase of probability of being a morning person.

***This effect is the transferred genetic effect, which describes the change of probability of being a morning person per unit increase of probability of being a morning person.

Table S9. Joint distribution of responses to two morningness surveys

		Survey Question S2					
		Morning person	Night person	Neither	It depends	I'm not sure	N/A
**Survey Question S1**	Early bird	4,295	122	192	493	37	5,232
	Night owl	187	8,147	285	1,013	87	7,193
	Neither	703	646	1,170	1,342	156	3,781
	N/A	31,777	42,196	8,290	16,526	1,577	117,735

This is a cross tabulation of the sample size for combinations of responses to the two morning person surveys (S1 and S2).
Survey Question S1: Are you naturally a night person or a morning person? **Response:** Night owl, Early bird, Neither.
Survey Question S2: Are you naturally a night person or a morning person? **Response:** Night person, Morning person, Neither, It depends, I'm not sure.

Table S10. Summary of newly identified significant loci in the GWAS of *chronotype*

Gene context	Marker name	Chromosome	Position	SNP quality	Alleles (A/B)	BAF	Effect for B allele	95% CI	P value
MSRA	rs2975734	8	10090097	0.9897	C/G	0.4331	0.023	[0.015, 0.03]	2.1×10^{-9}
PHACTR1	rs9357620	6	13170634	0.9763	C/T	0.7088	0.023	[0.015, 0.032]	1.7×10^{-8}

Table S11. Detailed analysis between BMI, depression and morning person

A. Analysis of morning person and BMI. Effect is in log odds ratio scale.

BMI category	Sample size	Effect	Standard error	P value
Reference: 18.5-25	33822	Model*: Morning person ~ BMI category*		
<18.5	1556	-0.1617	0.05309	0.002314
25-30	27523	0.03457	0.01637	0.03474
>30	17141	-0.1695	0.01913	7.812e-19
Reference: 18.5-25	33822	Model: *Morning person ~ BMI category + age*		
<18.5	1556	-0.03536	0.05636	0.5305
25-30	27523	-0.1864	0.01747	1.383e-26
>30	17141	-0.4404	0.02034	5.439e-104
Reference: 18.5-25	33822	Model: *Morning person ~ BMI category + sex*		
<18.5	1556	-0.2137	0.05331	6.088e-05
25-30	27523	0.1055	0.0167	2.732e-10
>30	17141	-0.1414	0.01923	1.937e-13
Reference: 18.5-25	33822	Model: *Morning person ~ BMI category + age + sex*		
<18.5	1556	-0.06836	0.05646	0.226
25-30	27523	-0.1396	0.01782	4.833e-15
>30	17141	-0.4179	0.02042	4.233e-93
Reference: 18.5-25	33822	Model: *Morning person ~ BMI category + age + sex + PC1-5*		
<18.5	1556	-0.06626	0.05653	0.2411
25-30	27523	-0.1403	0.01784	3.772e-15
>30	17141	-0.4202	0.02047	1.253e-93
Reference: 18.5-25	13397	Model: *Morning person ~BMI category + age + sex + PC1-5 + alcohol_abuse*		
<18.5	510	-0.06456	0.09393	0.4918
25-30	13391	-0.1667	0.02584	1.122e-10
>30	9366	-0.462	0.02817	1.917e-60
Reference: 18.5-25	18348	Model: *Morning person ~ BMI category + age + sex + PC1-5 +*		

		nicotine_abuse		
<18.5	839	-0.02625	0.07675	0.7323
25-30	15570	-0.1497	0.02384	3.403e-10
>30	10547	-0.4498	0.0266	3.941e-64
Reference: 18.5-25	10872	Model: *Morning person ~ BMI category + age + sex + PC1-5 + current_caffeine_use*		
<18.5	524	0.0532	0.097	0.5833
25-30	9491	-0.1314	0.03075	1.94e-05
>30	6680	-0.4136	0.03391	3.196e-34

B. Analysis of morning person and depression. Effect is in log odds ratio scale.

**Depression status**	**Sample size**	**Effect**	**Standard error**	**P value**
No	44379	Model*: Morning person ~ depression*		
Yes	16812	-0.4537	0.0188	1.069e-128
No	44379	Model: *Morning person ~ depression + age*		
Yes	16812	-0.4478	0.01963	3.517e-115
No	44379	Model: *Morning person ~ depression + sex*		
Yes	16812	-0.5179	0.01914	3.526e-161
No	44379	Model: *Morning person ~ depression + age + sex*		
Yes	16812	-0.4973	0.01996	4.968e-137
No	44379	Model: *Morning person ~ depression + age + sex + PC1-5*		
Yes	16812	-0.4968	0.02001	4.088e-136
No	26589	Model: *Morning person ~ depression + age + sex + PC1-5 + alcohol_abuse*		
Yes	10718	-0.5088	0.02458	3.407e-95
No	34342	Model: *Morning person ~ depression + age + sex + PC1-5 + nicotine_abuse*		
Yes	13498	-0.4903	0.02262	3.364e-104
No	18712	Model: *Morning person ~ depression + age + sex + PC1-5 + current_caffeine_use*		
Yes	7845	-0.4997	0.02983	5.608e-63

Table S12. The relationship between index SNP for known circadian genes and reported SNPs that are associated with circadian rhythm related phenotypes

Index SNP	Gene name	Related phenotypes	SNPs (r^2, P value for morningness GWAS)
rs55694368	*PER2*	familial advanced sleep phase syndrome	rs121908635 (N/A)
rs11121022	*PER3*	diurnal preference	rs228697 (r^2 = 0.08, P =5.3x10^{-5})
rs35833281	*HCRTR2*	cluster headache, narcolepsy	rs2653349 (r^2 = 0.25, P = 3.6x10^{-7})<br>rs3122169 (r^2 = 0.31, P = 1.8x10^{-6})
rs12927162	*TOX3*	restless leg syndrome	rs3104767 (r^2 = 1.2x10^{-4}, P = 0.27)
		breast cancer	rs3803662 (r^2 = 3.5x10^{-4}, P = 0.024)<br>rs4784227 (r^2 = 4.3x10^{-4}, P = 0.018)

In addition to literature review of our identified SNPs, we looked up genes in OMIM (*http://www.ncbi.nlm.nih.gov/omim*) for the SNP names for relevevant phenotypes. We summarized in the above table the r^2 between our index SNPs and these SNPs and the P values in our morning person GWAS for these SNPs.

Table S13. The associations of PCs with genetic risk, intermediate variables and outcomes in our Mendelian randomization analysis

A. MR using morning person genetic risk as an instrument variable to infer the causal relationship between morning person, depression and BMI.

Covariate	Effect	Standard error	P value
Model: Morning person genetic risk ~ age + sex +PC1-PC5 (n = 239,560)			
PC1	0.0156	0.0052	0.00273
PC2	0.0376	0.0111	0.000697
PC3	0.0174	0.0121	0.151
PC4	-0.0955	0.0191	5.73e-07
PC5	0.141	0.0211	2.25e-11
Model: BMI ~ age + sex +PC1-PC5 (n = 192,600)			
PC1	-5.48	0.239	7.5e-116
PC2	10.9	0.513	4.57e-100
PC3	1.28	0.561	0.0223
PC4	0.0698	0.883	0.937
PC5	4.63	0.954	1.19e-06
Model: Depression ~ age + sex +PC1-PC5 (n =112,619)			
PC1	-1.05	0.15	2.97e-12
PC2	3.3	0.335	6.42e-23
PC3	5.54	0.37	1.29e-50
PC4	0.942	0.577	0.103
PC5	0.699	0.559	0.211

B. MR using BMI genetic risk as an instrument variable to infer the causal relationship between BMI and morning person.

Covariate	Effect	Standard error	P value
Model: BMI genetic risk ~ age + sex + PC1-PC5 (n = 239,560)			
PC1	-0.547	0.0197	2.74e-169
PC2	-0.00158	0.0421	0.97
PC3	-0.0615	0.046	0.18
PC4	-0.387	0.0724	8.91e-08
PC5	0.295	0.0801	0.00023
Model: morning_person ~ age + sex + PC1-PC5 (n = 100,585)			
PC1	0.437	0.138	0.00159
PC2	1.3	0.299	1.37e-05
PC3	-0.72	0.326	0.0274
PC4	-2.65	0.511	2.21e-07
PC5	-0.935	0.534	0.0798

Table S14. Exploring pleiotropy for morning person and BMI genetic risk

A. Morning person genetic risk

Other phenotype	Sample size	Effect	Standard error	P value
Model: other phenotype ~ morning_person genetic risk + age + sex + PC1-PC5				
anxiety	98,639	-0.0232	0.0679	0.733
alcohol_abuse	76,353	0.0812	0.0795	0.307
current_caffeine_use	50,992	5.18	8.27	0.531
sweety_v_salty	78,350	0.147	0.0547	0.00705
Average daily sleep < 8 hours	40,458	-0.171	0.0809	0.035
Model: other phenotype ~ BMI + age + sex + PC1-PC5				
anxiety	88,106	0.0225	0.00157	2.37e-46
alcohol_abuse	70,459	-0.00632	0.002	0.00159
current_caffeine_use	49,048	1.08	0.195	2.83e-08
sweety_v_salty	70,339	0.00891	0.00139	1.49e-10
Average daily sleep < 8 hours	37,621	0.021	0.00196	6.74e-27
Model: other phenotypes ~ depression + age + sex + PC1-PC5				
anxiety	95023	2.4	0.0209	$<1x10^{-200}$
alcohol_abuse	71397	0.704	0.0229	2.03e-206
current_caffeine_use	44965	-1.65	2.63	0.532
sweety_v_salty	51672	0.217	0.0202	6.87e-27
Average daily sleep < 8 hours	37063	-0.287	0.0247	2.98e-31

B. BMI genetic risk

Other phenotype	Sample size	Effect	Standard error	P value
Model: other phenotype ~ BMI genetic risk + age + sex + PC1-PC5				
anxiety	96,929	-0.0124	0.0183	0.499
alcohol_abuse	76,263	-0.0295	0.0216	0.172
current_caffeine_use	50,853	10.5	2.17	1.28e-06
sweety_v_salty	77,024	0.13	0.0146	6e-19
Average daily sleep < 8 hours	39,846	0.0315	0.0215	0.143
Model: other phenotype ~ morning_person + age + sex + PC1-PC5				
anxiety	57,929	-0.448	0.0239	4.84e-78
alcohol_abuse	44,425	-0.205	0.0269	2.79e-14
current_caffeine_use	33,261	18.2	2.77	4.53e-11
sweety_v_salty	58,961	0.0313	0.0168	0.0631
Average daily sleep < 8 hours	30,153	-0.0441	0.025	0.0775

Table S15. Details of MR analysis, with consideration of PCs and other phenotype

A. MR of BMI using morning person genetic risk as an instrument variable with adjustments of PCs and other phenotype

Covariate	Effect	Standard error	P value
Model: ***BMI*** *~ Morning person genetic risk + age + sex (n* = 205,241*)*			
**Risk**	**-0.0755**	**0.0901**	**0.402**
Model: ***BMI*** *~ Morning person genetic risk + age + sex +PC1-PC5 (n* = 191,822*)*			
**Risk**	**-0.0731**	**0.093**	**0.432**
PC1	-5.48	0.24	1.86e-115
PC2	10.9	0.514	5.31e-100
PC3	1.28	0.563	0.0232
PC4	0.158	0.888	0.859
PC5	4.59	0.957	1.57e-06
Model: ***BMI*** *~ Morning person genetic risk + age + sex + PC1-PC5 + sweety_v_salty* (n =)			
**Risk**	**-0.2849**	**0.1573**	**0.07015**
sweety_v_salty	0.2707	0.04162	7.796e-11
PC1	-5.703	0.4312	6.962e-40
PC2	11.29	0.9029	8.266e-36
PC3	1.571	1.005	0.118
PC4	2.744	1.549	0.07643
PC5	3.097	1.606	0.05376

B. MR of depression using morning person genetic risk as an instrument variable with adjustments of PCs and other phenotype

Covariate	Effect	Standard error	P value
Model: **Depression** *~ Morning person genetic risk + age + sex (n* = 116,818*)*			
**Risk**	**-0.0853**	**0.0521**	**0.101**
Model: **Depression** *~ Morning person genetic risk + age + sex +PC1-PC5 (n* = 112,244*)*			
**Risk**	**-0.0868**	**0.0533**	**0.103**
PC1	-1.05	0.15	2.38e-12
PC2	3.3	0.335	8.01e-23
PC3	5.55	0.371	1.97e-50
PC4	0.883	0.582	0.129
PC5	0.713	0.561	0.203
Model: **Depression** *~ Morning person genetic risk + age + sex +PC1-PC5 + sweety_v_salty*			

(n = 50,947)			
**Risk**	**-0.1771**	**0.07871**	**0.02441**
sweety_v_salty	0.1846	0.0206	3.233e-19
PC1	-0.9505	0.2286	3.202e-05
PC2	3.604	0.4968	4.008e-13
PC3	6.047	0.56	3.445e-27
PC4	1.043	0.8648	0.2276
PC5	-0.5283	0.8112	0.5149

C. MR of morning person using BMI genetic risk as an instrument variable with adjustment of PCs and other phenotype

Covariate	Effect	Standard error	P value
Model: ***morning_person*** *~ risk + age + sex (n =* 105,585*)*			
**Risk**	**0.00866**	**0.0131**	**0.509**
Model: ***morning_person*** *~ risk + age + sex + PC1-PC5 (n =* 100,270*)*			
**Risk**	**0.0151**	**0.0135**	**0.263**
PC1	0.446	0.139	0.0013
PC2	1.35	0.3	6.7e-06
PC3	-0.758	0.327	0.0205
PC4	-2.71	0.514	1.32e-07
PC5	-0.901	0.536	0.0927
Model: morning_person ~ risk + age + sex + PC1-PC5 + current_caffeine_use (n = 33,044)			
**Risk**	**0.007387**	**0.02359**	**0.7541**
current_caffeine_use	8.191e-05	4.739e-05	0.08394
PC1	0.2881	0.2667	0.28
PC2	1.246	0.5479	0.02293
PC3	0.4705	0.6339	0.4579
PC4	-3.249	0.9945	0.001087
PC5	-0.8734	0.9388	0.3522

Table S16. HaploReg results for the functional annotations of significant SNPs

A. Screen shot of the summarized HaploReg results.

chr	pos (hg19)	LD (r^2)	LD (D')	variant	Ref	Alt	AFR freq	AMR freq	ASN freq	EUR freq	SiPhy cons	Promoter histone marks	Enhancer histone marks	DNAse	Proteins bound	eQTL tissues	Motifs changed	GENCODE genes	dbSNP func annot
1	182549729	0	0	rs12736689	T	C	0.09	0.02	0.00	0.02							8 altered motifs	RNASEL	intronic
6	153135339	0	0	rs9479402	T	C	0.09	0.06	0.28	0.01							4 altered motifs	RP1-200K18.1	
2	239317692	0	0	rs55694368	G	T	0.00	0.04	0.05	0.11			H1	5 cell types			5 altered motifs	3.6kb 5' of U6	
6	55021561	0	0	rs35833281	G	C	0.02	0.15	0.02	0.21							8 altered motifs	17kb 5' of HCRTR2	
17	17398278	0	0	rs11545787	G	A	0.11	0.20	0.07	0.24		6 cell types		7 cell types			Myf	RASD1	3'-UTR
1	7836659	0	0	rs11121022	A	C	0.16	0.34	0.26	0.39							CACD,STAT,ZBTB7A	VAMP3	intronic
13	77577027	0	0	rs9565309	T	C	0.00	0.09	0.13	0.04				16 cell types	CTCF,RAD21,SMC3			FBXL3	
2	198874006	0	0	rs1595824	C	T	0.31	0.40	0.24	0.53								PLCL1	intronic
1	150234657	0	0	rs34714364	G	T	0.00	0.09	0.00	0.15			HMEC, NHEK				11 altered motifs	CA14	synonymous
7	102436907	0	0	rs3972456	G	A							Huvec	8 cell types	GATA2		Pax-2,RREB-1,SIX5	FAM185A	intronic
18	31675680	0	0	rs12965577	A	G	0.09	0.37	0.44	0.38							Sox,TEF-1	NOL4	intronic
16	52684916	0	0	rs12927162	A	G	0.02	0.24	0.00	0.26							Pou2f2	RP11-297L17.1	
1	77726241	0	0	rs10493596	C	T	0.26	0.21	0.23	0.23							ERalpha-a	21kb 5' of AK5	
7	96457119	0	0	rs2948276	A	G	0.64	0.19	0.09	0.17				H9ES	CTCF,RAD21		4 altered motifs	112kb 3' of Metazoa_SRP	
12	38726137	0	0	rs6582618	A	G	0.52	0.66	0.85	0.53							Hlx1,Mef2,TATA	8.4kb 3' of ALG10B	
8	10090097	0	0	rs2975734	C	G	0.26	0.32	0.19	0.41							AP-3,RXRA,TCF11::MafG	MSRA	intronic
6	13170634	0	0	rs9357620	T	C	0.14	0.17	0.42	0.31							AP-1	PHACTR1	intronic

Haploreg results for the 17 significant SNPs (15 from *morning person* GWAS and 2 from *chronotype* GWAS).

B. Links for detailed HaploReg functional annotation.

SNP name	Link
rs12736689	http://www.broadinstitute.org/mammals/haploreg/detail_v2.php?query=&id=rs12736689
rs9479402	http://www.broadinstitute.org/mammals/haploreg/detail_v2.php?query=&id=rs9479402
rs55694368	http://www.broadinstitute.org/mammals/haploreg/detail_v2.php?query=&id=rs55694368
rs35833281	http://www.broadinstitute.org/mammals/haploreg/detail_v2.php?query=&id=rs35833281
rs11545787	http://www.broadinstitute.org/mammals/haploreg/detail_v2.php?query=&id=rs11545787
rs11121022	http://www.broadinstitute.org/mammals/haploreg/detail_v2.php?query=&id=rs11121022
rs9565309	http://www.broadinstitute.org/mammals/haploreg/detail_v2.php?query=&id=rs9565309
rs1595824	http://www.broadinstitute.org/mammals/haploreg/detail_v2.php?query=&id=rs1595824
rs34714364	http://www.broadinstitute.org/mammals/haploreg/detail_v2.php?query=&id=rs34714364
rs3972456	http://www.broadinstitute.org/mammals/haploreg/detail_v2.php?query=&id=rs3972456
rs12965577	http://www.broadinstitute.org/mammals/haploreg/detail_v2.php?query=&id=rs12965577
rs12927162	http://www.broadinstitute.org/mammals/haploreg/detail_v2.php?query=&id=rs12927162
rs10493596	http://www.broadinstitute.org/mammals/haploreg/detail_v2.php?query=&id=rs10493596
rs2948276	http://www.broadinstitute.org/mammals/haploreg/detail_v2.php?query=&id=rs2948276
rs6582618	http://www.broadinstitute.org/mammals/haploreg/detail_v2.php?query=&id=rs6582618
rs2975734	http://www.broadinstitute.org/mammals/haploreg/detail_v2.php?query=&id=rs2975734
rs9357620	http://www.broadinstitute.org/mammals/haploreg/detail_v2.php?query=&id=rs9357620
rs12736689	http://www.broadinstitute.org/mammals/haploreg/detail_v2.php?query=&id=rs12736689
